# Glucose deprivation impairs hypoxia-inducible factor-1α synthesis

**DOI:** 10.1007/s12672-024-01484-1

**Published:** 2024-10-28

**Authors:** Mia Hubert, Sarah Stuart, Michael Ohh

**Affiliations:** 1 Department of Laboratory Medicine & Pathobiology, Faculty of Medicine, University of Toronto, 1 King’s College Circle, Toronto, ON M5S 1A8 Canada; 2 Department of Biochemistry, Faculty of Medicine, University of Toronto, 1 King’s College Circle, Toronto, ON M5S 1A8 Canada

## Abstract

Hypoxia-inducible factors (HIFs) are key transcriptional mediators of the hypoxic response and are implicated in oncogenesis. HIFα is regulated by a well-characterized, oxygen-dependent degradation pathway involving the von Hippel Lindau (VHL) tumor suppressor protein. However, comparatively little is known about HIFα regulation at the translational level, particularly under cellular stress. There is evidence that HIFα expression not only responds to changes in oxygen tension, but also nutrient availability. In this study, we monitored global translation rates, ATP levels and HIF1α synthesis rates in response to glucose starvation or glycolysis inhibition. We found that both global and HIF1α-specific translation rates decline under glucose deprivation that is concomitant with ATP reduction. These results are in contrast with previous reports showing preferential HIF1α synthesis despite global translation suppression under hypoxia and suggest that a glucose requirement in cellular metabolism is associated with HIF1α translation.

**Supplementary Information:**

The online version contains supplementary material available at 10.1007/s12672-024-01484-1.

## Introduction

ATP is the energy currency that powers diverse cellular reactions. While ATP can be generated via the breakdown of various nutrients including glucose, amino acids, and lipids, the bulk of ATP production occurs via mitochondrial oxidative phosphorylation [1]. This process requires oxygen. However, cellular oxygen levels may be insufficient in ischemic tissues, solid tumors, or at high altitudes [2, 3]. The adaptive response to hypoxia consists of an evolutionarily conserved molecular pathway involving hypoxia inducible factors (HIFs) [4]. HIFs are transcription factors consisting of a constitutively expressed β subunit and an oxygen-responsive α subunit [5]. Oxygen-dependent HIFα regulation occurs via hydroxylation of conserved proline residues within the oxygen-dependent degradation domain (ODD) by prolyl hydroxylase domain (PHD) enzymes [6]. Hydroxylated HIFα is recognized by the von Hippel-Lindau protein (pVHL), which promotes polyubiquitination and rapid proteasomal degradation under normal oxygen conditions [7, 8]. Under hypoxia, hydroxylation of HIFα declines. Following stabilization, dimerization with HIF1β, and nuclear translocation, HIF guides transcription of target genes via recognition of enhancer sequences known as hypoxia-responsive elements (HREs) [9].

The two best-studied HIFα paralogs include HIF1α and HIF2α. While both undergo similar regulation by the PHD/VHL pathway and both recognize HREs, they differ in terms of temporal regulation and target genes [10]. HIF1α accumulates rapidly but declines under prolonged hypoxia (> 24 h) and is thus the main mediator of acute hypoxia [10, 11]. The response to chronic hypoxia is regulated by HIF2α, whose levels rise slowly but are sustained at prolonged timepoints [11]. Many genes are targets of both HIF1α and HIF2α, including vascular endothelial growth factor A (*VEGFA*), adrenomedullin (*ADM*), and glucose transporter 1 (*SLC2A1*) [12]. However, each paralog regulates a unique set of genes. Notably, many glycolytic genes are upregulated by HIF1α only [13]. As a result, HIF1α transcriptional activity is crucial in the ‘metabolic switch’ to glycolysis under hypoxia.

Dysregulation of HIFα may lead to inappropriate activation of the hypoxic response and can contribute to disease. For instance, mutations in *VHL* result in impaired HIFα degradation, leading to a hereditary cancer syndrome known as VHL disease, characterized by clear cell renal cell carcinoma, hemangioblastoma, paraganglioma, and polycythemia [14, 15]. Additionally, mutations in *PHD2* and *EPAS1* (HIF2α) can lead to some of the phenotypes of VHL disease [15]. Other oncogenic mutations, such as in p53, Myc, Rb, and Ras, can lead to abnormal HIFα levels [16, 17]. HIF activity in cancer can contribute to tumorigenic metabolic adaptations, epithelial-mesenchymal transition, metastasis, and resistance to chemotherapy or radiotherapy [18–20].

There is evidence that HIFα can be impacted by cellular stimuli other than hypoxia. For example, accumulations of metabolites including succinate, fumarate, lactate, and pyruvate have been found to impair PHD activity, leading to HIFα stabilization at normal oxygen levels [21–24]. Activation of signal transduction pathways have also been shown to impact HIF1α at multiple regulatory levels. For instance, downstream effectors of the phosphoinositide-3-kinase and protein kinase B (PI3K/Akt) pathway, including mammalian target of rapamycin (mTOR) and glycogen synthase kinase-3β (GSK-3β), may interfere with VHL-dependent degradation or promote VHL-independent degradation, respectively [25, 26]. HIF1α synthesis also appears to be dependent on mTORC1, a key regulator of translation initiation [27, 28]. Endoplasmic reticulum (ER) stress has also been shown to impair HIF1α synthesis via PKR-like endoplasmic reticulum kinase (PERK) activation by the unfolded protein response (UPR) [29].

Limited blood flow in hypoxic tissues can lead to poor nutrient delivery, including glucose, to cells [30]. Additionally, glucose consumption is accelerated in hypoxic cells [31]. As a result, HIF1α expression often coincides with low glucose availability. The impact of glucose deprivation on HIF1α expression has been explored in the past. In general, most groups have found that glycolysis inhibition or glucose starvation decreases HIF1α levels [32–37]. Interestingly, more recent reports have found the opposite [38, 39]. However, there is no strong consensus on the mechanism behind HIF1α regulation by glucose deprivation, with some groups suggesting translational or post-translational mechanisms.

Glycolysis inhibition or glucose starvation impairs global translation rates via inhibition of mTORC1 or activation of the PERK-mediated ER stress pathway [40–42]. Additionally, short-lived proteins appear to be particularly sensitive to translational downregulation by glucose deprivation [42]. Given the short half-life of HIF1α (approximately five minutes at normal oxygen conditions) [5] and evidence that HIF1α synthesis responds to regulation by mTORC1/PERK, we focused on the potential translational effects of glucose deprivation on HIF1α. Using luminescence-based cell viability assays and puromycin incorporation assays, we explored how ATP levels, global translation rates, and HIF1α-specific translation rates respond to glycolysis inhibition or glucose starvation. We found that in general, glycolysis inhibition with 2-deoxyglucose (2-DG) or glucose starvation impairs both global and HIF1α-specific translation rates.

## Materials and methods

### Antibodies

The following antibodies were used for immunoblotting and immunoprecipitation: HIF1α (1:1000, #36169) and rabbit (DA1E) mAb IgG isotype control (#3900) antibodies were purchased from Cell Signaling Technology (Danvers, MA). Puromycin clone 12D10 (1:5000, MABE343) and vinculin (1:1000, V9264) antibodies were purchased from Sigma-Adrich (Oakville, ON). Primary antibodies were diluted in 5% bovine serum albumin (BSA) with 0.02% sodium azide for immunoblotting.

### Cell lines

Wild-type (WT) human epithelial kidney HEK293A cells were obtained from American Type Culture Collection (ATCC). Homozygous *VHL* knockout (-/-) HEK293A cells were generated via the clustered regularly interspaced short palindromic repeats (CRISPR) method. CRISPR guides targeting *VHL* were selected using the CRISPOR design tool [43]. Oligonucleotides (forward primer: 5’–caccgCATACGGGCAGCACGACG CG –3’; reverse primer: 5’–aaacCGCGTCGTGCTGCCCGT ATGc–3’) were phosphorylated and annealed, then ligated to *Bbs*I-digested pX330-U6-Chimeric_BB-CBh-hSpCas9 (a gift from Feng Zhang; Addgene plasmid # 42230; http://n2t.net/addgene:42230; RRID:Addgene 42230). The HEK293A-*VHL*-/- *cell* line was generated by transfecting HEK293A cells with pX330-*VHL* guide RNA using Lipofectamine 2000, as per manufacturer’s instructions. Single-cell clones were isolated by seeding into 96-well plates at a density of ~ 0.2 cells/well. Gene disruption was confirmed by Western blotting for VHL. RCC4 cells (VHL-deficient, human renal cell carcinoma cell line) were generously provided by Dr. Wafik S. El-Deiry (Penn State Milton S. Hershey Medical Center).

### Cell culture

Cells were cultured in Dulbecco’s modified Eagle’s Medium (DMEM, 4.5 g/L glucose, with L-glutamine, Wisent 319-015) supplemented with 10% fetal bovine serum (FBS, Wisent 090-150) and 1% solution containing 10,000 IU/mL penicillin and 10,000 μg/mL streptomycin (Wisent 450-201). Cells were grown in a humidified incubator at 37 ℃ with 5% CO_2_ and atmospheric oxygen (⁓19.95% O_2_) and passaged when approximately 80–90% confluent.

HEK293A and RCC4 cells were seeded 1 day prior to experiments to ensure 80–90% confluency. Spent media was removed and cells were washed with phosphate-buffered saline (PBS). Fresh supplemented DMEM was added to cells, alongside the following treatments and final concentrations: dimethyloxalylglycine (DMOG, 1 mM, Cayman Chemicals 71210), 2-deoxy-D-glucose (2-DG, 1–100 mM, BioShop DXG498.5), cycloheximide (CHX, 20 μg/mL, Millipore Sigma C7698), D-( +)-mannose (10 mM, Millipore Sigma M6020), and sodium pyruvate (1 or 5 mM, Millipore Sigma P5280). Cells were incubated in a humidified chamber for the indicated times at atmospheric oxygen or hypoxia (1% O_2_) with 5% CO_2_.

### Surface sensing of translation (SUnSET) assay

To measure protein synthesis non-radioactively in cultured cells, a surface sensing of translation (SUnSET) assay was performed [44]. Puromycin dihydrochloride (10 μg/mL, Sigma-Aldrich P9620) was added directly into culture media either 10 min or 1 h prior to cell collection for immunoblotting or immunoprecipitation experiments, respectively.

### Preparation of whole-cell lysates

Prior to cell collection, culture media was removed, and cells were washed three times with pre-chilled PBS. For immunoblotting, cells were collected directly in the culture dish via manual scraping in EBC lysis buffer (50 mM Tris, 120 mM NaCl, 0.5% NP-40) supplemented with 1 mM DMOG and 1 × protease inhibitor cocktail (BioShop PIC002.1). Larger culture dishes were used for immunoprecipitation experiments, and thus cells were collected via addition of 0.25% Trypsin-ethylenediaminetetraacetic acid (EDTA) (Wisent 325-043) to detach adherent cells. Following neutralization of Trypsin with DMEM, the cell suspension was centrifuged for 5 min at 1100 RPM. The cell pellet was washed two additional times with PBS, followed by resuspension in EBC lysis buffer. Cells under hypoxic treatments were collected within a hypoxic glovebox station to minimize reoxygenation. For all experiments, the cell-lysis buffer suspension was transferred to a sterile microcentrifuge tube and sonicated for 5 s at 30% intensity 3–5 times, depending on cell pellet size. Following centrifugation for 10 min at 14,800 RPM at 4℃, supernatants were transferred to a fresh microcentrifuge tube and stored at −80 ℃. Protein concentrations within whole cell lysates were quantified via Bradford assay. For immunoblotting, samples were prepared to contain equal total protein concentrations prior to boiling in sample buffer (62.5 mM Tris, 10% glycerol, 2% SDS, 0.01% bromophenol blue) for five minutes to denature proteins.

### Immunoprecipitation

For immunoprecipitation experiments, samples were prepared to contain equal total protein concentrations within 500 μL of EBC lysis buffer. A 5% input was removed from each sample and processed for immunoblotting as described below. For RCC4 cells, a higher total protein concentration was used for cycloheximide-treated samples to ensure equal pull-down of HIF1α. A non-adjusted and adjusted input was included in the 5% input blots. Lysates were incubated with 200 ng of HIF1α primary antibody or control rabbit IgG antibody overnight at 4 ℃ with rotation. Fifty μL of protein A Sepharose bead slurry (Repligen, IPA-400HC-10-2500) pre-blocked with 100 μg/mL BSA was added to lysates for an additional hour at 4 ℃ with rotation. Lysates were centrifuged for 30 s at 5000 RPM, the supernatant was removed, and pelleted beads were washed with NETN buffer (20 mM Tris, 100 mM NaCl, 1 mM EDTA. 0.5% NP-40). This process was repeated for four additional washes, followed by elution of immunoprecipitated proteins from the beads via boiling in sample buffer for five minutes.

### Immunoblotting

Proteins within whole cell lysates, immunoprecipitation samples, and input samples were resolved via gel electrophoresis (7.5% acrylamide, 75 min at 150 V) and transferred to polyvinylidene fluoride (PVDF) membranes via wet electrophoretic transfer (120 min at 250 mA). Membranes were blocked with 5% skim milk prepared in Tris-buffered saline with Tween (TBST) for one hour at room temperature, followed by incubation with the indicated primary antibodies overnight at 4 ℃. Membranes were washed three times with TBST, followed by incubation with the corresponding HRP-conjugated secondary antibody (1:30,000 in 5% skim milk/TBST) for one hour, and three more TBST washes. To detect proteins of interest, membranes were incubated with varying enhanced chemiluminescence solutions depending on the antibody signal strength (SuperSignal^™^ West Dura Extended Duration Substrate or Femto Maximum Sensitivity Substrate, ThermoFisher Scientific 34075 and 34096, respectively) and detected with a ChemiDoc^™^ MP Imaging System (BioRad).

### ATP quantification

Cells were seeded in 96-well plates 1 day prior to experiments to ensure 80–90% confluency. Spent media was removed and replaced with 100 μL fresh media containing the corresponding treatments, as described above. Following four hours of treatment, cells were processed with the CellTiter-Glo^®^ Luminescent Cell Viability Assay (Promega G7571) as per the manufacturer’s protocol. Mixtures were transferred to white polystyrene 96-well plates and luminescence was read with a luminescent plate reader. Luminescence values for each treatment sample were corrected for background luminescence using readouts from media-only samples. Average luminescence and standard deviation for each treatment type was determined from three technical replicates (or six for untreated, DMOG-only, or hypoxia-only samples) and normalized to the untreated samples (set to 100%). Unpaired, two-tailed t-tests were conducted to identify statistical differences between individual and combination treatments. Graphs were generated using GraphPad Prism version 10.0.0 for Windows, GraphPad Software, Boston, Massachusetts, USA, www.graphpad.com.

## Results

### Glucose deprivation suppresses HIF1α accumulation in DMOG- or hypoxia-treated HEK293A cells

**Fig. 1:**
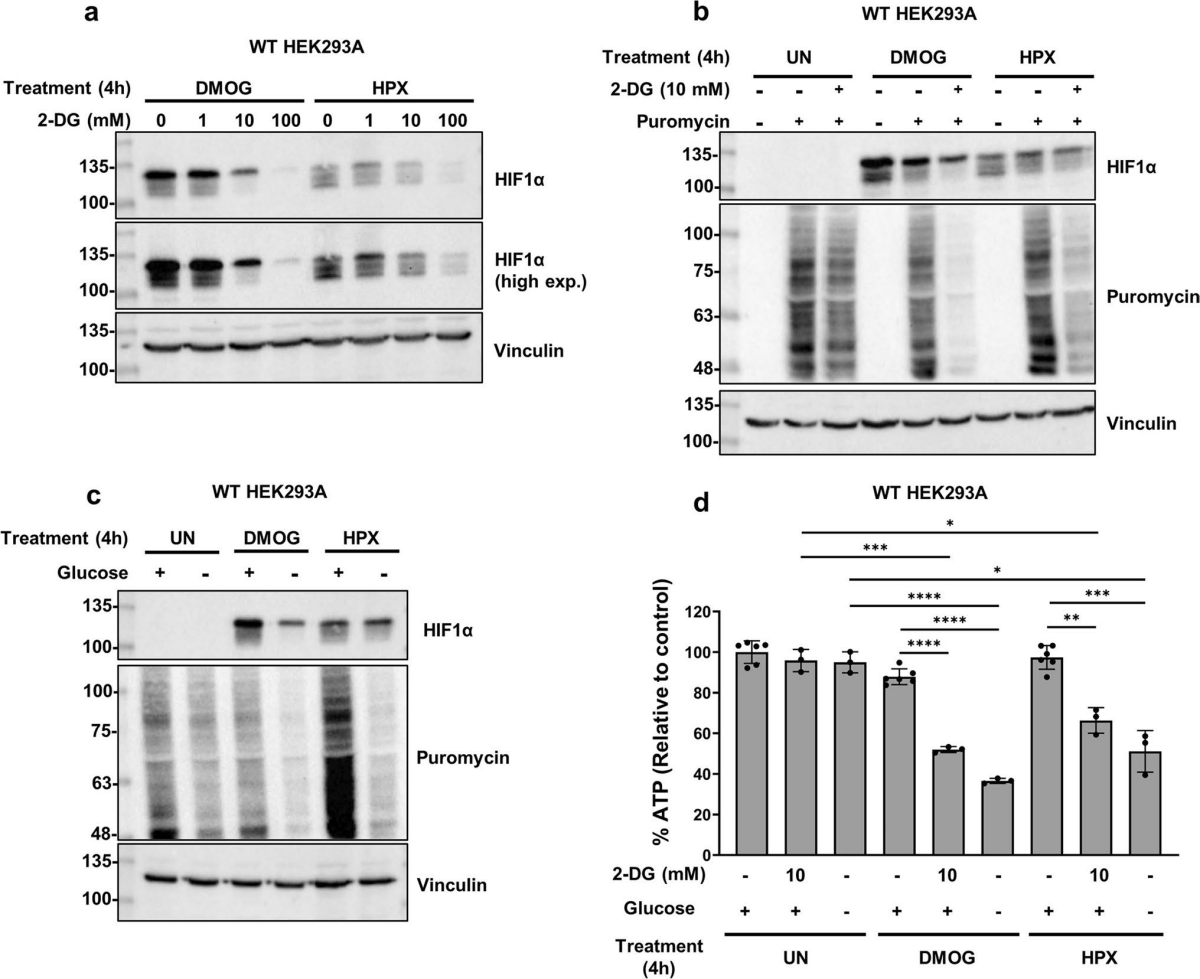
Glycolysis inhibition and glucose starvation impair HIF1α accumulation in DMOG- or hypoxia-treated HEK293A cells. In all experiments, WT HEK293A cells were treated with DMOG (1 mM) or hypoxia (HPX, 1% O_2_) for four hours to stabilize HIF1α. Glycolysis inhibition was tested via addition of 2-DG (1–100 mM in (**a**) and 10 mM in (**b**)). **c** Glucose starvation was tested via treatment with glucose-containing (+ , 25 mM) or glucose-free (−, 0 mM) DMEM. (**b**, **c**) Puromycin (10 μg/mL) was added to cells 10 min before collection to label actively-translating peptides. Whole cell lysates were processed for immunoblotting and blots were probed with the indicated antibodies. Blots are representative of three independent experiments. **d** WT HEK293A cells were exposed to the indicated treatments for four hours and processed via luminescent cell viability assay to assess relative ATP content. Average luminescence was calculated from three technical replicates (or six replicate samples for the untreated, DMOG only, or hypoxia only treatments) and normalized to the untreated average values (set to 100%). Values are displayed as normalized average ± SD. *p < 0.01, **p < 0.001, *** p < 0.0001, and ****p < 0.00001 (two-tailed unpaired t-test)

To determine if glycolysis inhibition impacts HIF1α expression, human epithelial kidney HEK293A cells were treated with hypoxia or DMOG to promote HIF1α stabilization in addition to increasing concentrations of 2-DG. DMOG is an analog of α-ketoglutarate that is widely used in hypoxia research to induce HIFα stabilization via PHD inhibition [45]. By 4 h of treatment, HIF1α accumulation was impaired by 2-DG treatment in a dose-dependent manner (Fig. 1a). A consistent effect on HIF1α was seen with 10 mM 2-DG (Fig. 1b), a dose within the range used in other in vitro reports. Additionally, HIF1α levels were assessed in response to glucose-free media treatment. Glucose starvation also impaired HIF1α accumulation by 4 h (Fig. 1c). For both 2-DG and glucose-free media treatments, the effect on HIF1α appeared to be greater in DMOG-treated samples compared to hypoxia samples.

### DMOG or hypoxia potentiate the effects of glucose deprivation on ATP levels and global translation rates

Glucose starvation or glycolysis inhibition has been shown to suppress ATP levels and global translation rates. As a result, we monitored global translation rates and ATP levels following 2-DG or glucose-free media treatment via SUnSET method and luminescent cell viability assays, respectively. The relative levels of puromycin-labelled peptides reflect the rates of protein synthesis across samples. By 4 h, both 10 mM 2-DG and glucose-free media treatment suppressed global translation rates slightly compared to basal levels (Fig. 1b, c). However, a stronger decline in translation rates was noted in samples treated with DMOG or hypoxia in combination with glucose deprivation than any treatment alone. A similar pattern was observed with ATP levels by 4 h of treatment (Fig. 1d). 2-DG or glucose-free media alone did not significantly decrease ATP levels compared to untreated samples. However, ATP levels decreased significantly more with glucose deprivation in combination with DMOG or hypoxia compared to individual treatments.

### Total HIF1α levels are relatively unaffected by glucose deprivation alone in VHL-deficient cell lines

Based on our experimentation in DMOG- or hypoxia-treated HEK293A cells, it is unclear if the suppression of HIF1α is from glucose deprivation alone or the combination of treatments. However, treatment with hypoxia or PHD inhibition is required to stabilize HIF1α in HEK293A cells harbouring WT pVHL. To determine if 2-DG or glucose starvation alone can have an effect on HIF1α, we generated *VHL-/-* HEK293A cells via CRISPR knockout, which have stable HIF1α regardless of oxygen concentration. Additionally, a VHL-deficient renal cell carcinoma cell line (RCC4) was also tested. Cells were treated with 10 mM 2-DG or glucose-free media alone or in combination with DMOG or hypoxia for 4 or 24 h. Finally, cycloheximide treatment was included as a positive control for translation inhibition. ATP levels and global translation rates were assessed as in previous experiments.

**Fig. 2:**
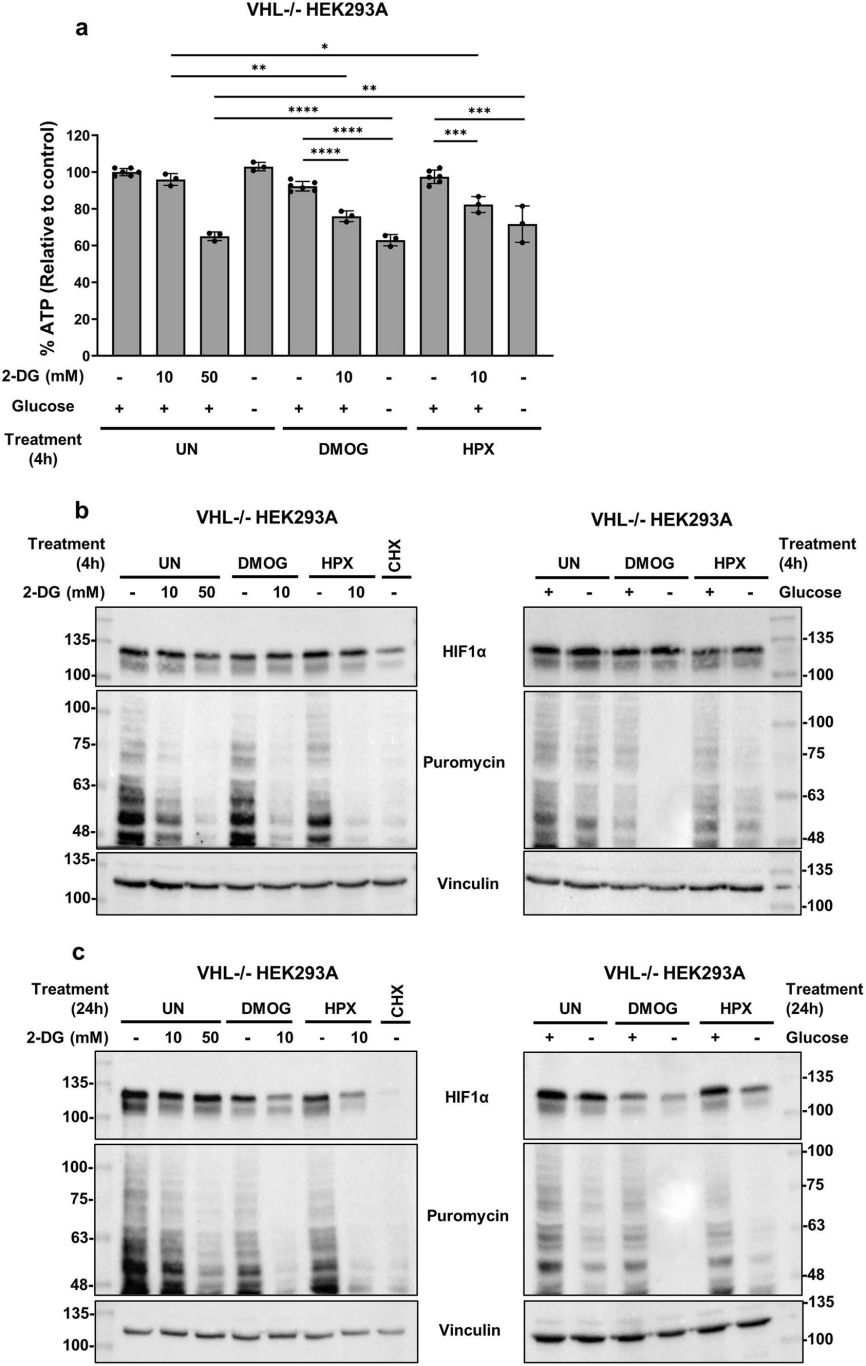
Glucose deprivation alone does not appear to substantially suppress HIF1α levels by 24 h in *VHL-/-* HEK293A cells. In all experiments, *VHL-/-* HEK293A cells were treated with DMOG (1 mM), hypoxia (HPX, 1% O_2_), or cycloheximide (CHX, 20 μg/mL). Glycolysis inhibition was tested via addition of 2-DG (10 or 50 mM). Glucose starvation was tested via treatment with glucose-containing (+ , 25 mM) or glucose-free (−, 0 mM) DMEM. **a** After four hours of treatment, cells were processed via luminescent cell viability assay to assess relative ATP content. Average luminescence was calculated from three technical replicates (or six replicate samples for the untreated, DMOG only, or hypoxia only treatments) and normalized to the untreated average values (set to 100%). Values are displayed as normalized average ± SD. *p < 0.01, **p < 0.001, ***p < 0.0001, and ****p < 0.00001 (two-tailed unpaired t-test). After 4 (**b**) or 24 (**c**) hours of treatment, puromycin (10 μg/mL) was added to cells 10 min before collection. Whole cell lysates were processed via immunoblotting and blots were probed for the indicated antibodies. Blots are representative of three independent experiments

In *VHL-/-* HEK293A cells, ATP levels and translation rates declined in a similar fashion as in WT HEK293A cells (Fig. 2a). However, ATP levels declined approximately 20% less than WT HEK293A cells with the combination treatments. Higher-dose 2-DG resulted in a stronger effect on ATP and translation compared to the 10 mM dose. By 4 h, HIF1α levels were relatively unaffected by any treatment in *VHL-/-* HEK293A cells (Fig. 2b). Cycloheximide successfully suppressed HIF1α levels, indicating that translation inhibition can impact total HIF1α levels by 4 h. By 24 h, 2-DG treatment or glucose starvation minimally impacted HIF1α levels despite a sustained suppression of global translation (Fig. 2c). However, combination with DMOG or hypoxia successfully suppressed HIF1α levels by 24 h. These results suggest that the suppression of HIF1α accumulation in HEK293A cells is not solely due to glucose deprivation, but rather the combination with DMOG or hypoxia.

**Fig. 3:**
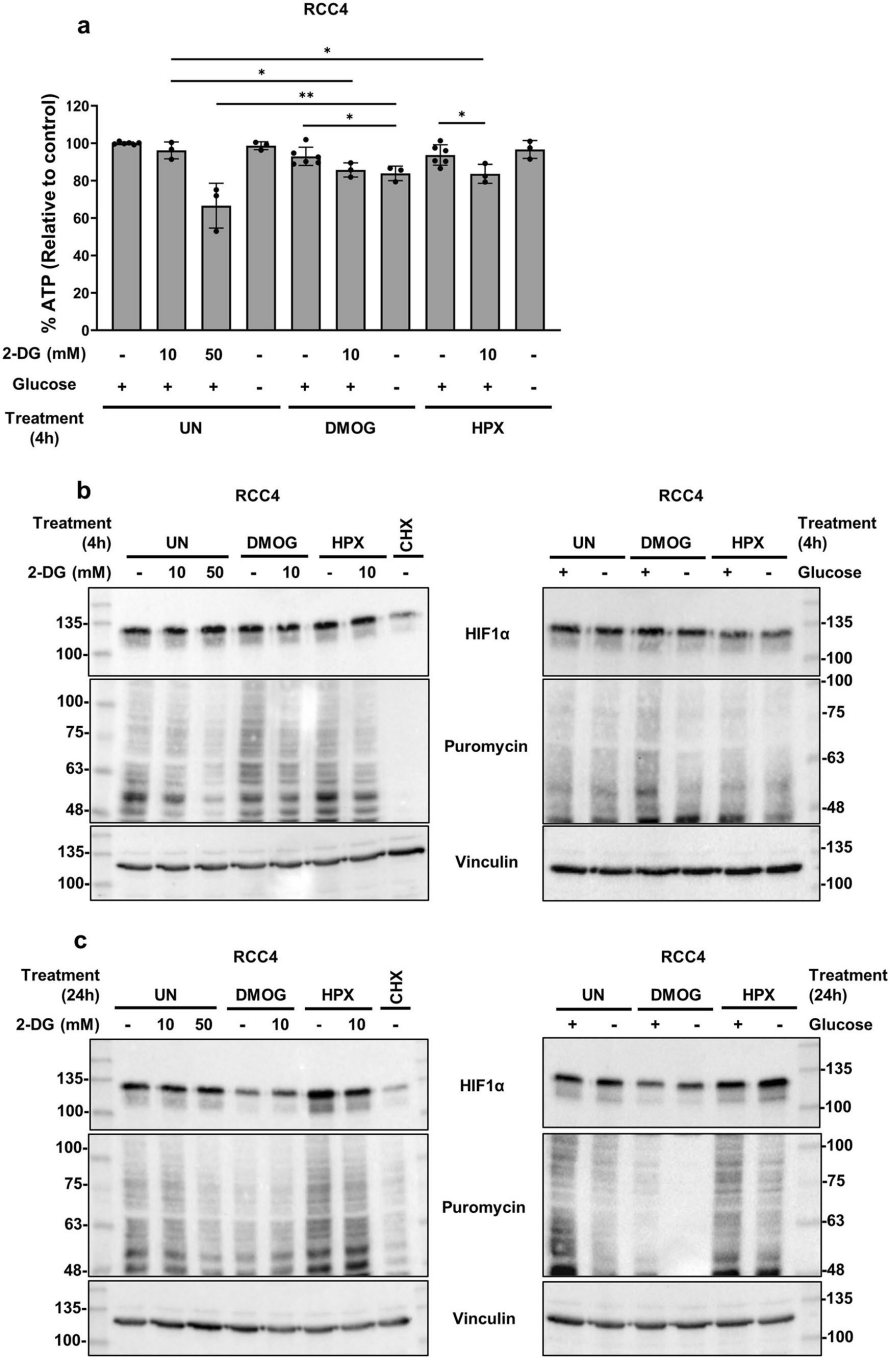
DMOG treatment, but not glucose deprivation, appears to suppress HIF1α expression by 24 h in RCC4 cells. In all experiments, RCC4 cells were treated with DMOG (1 mM), hypoxia (HPX, 1% O_2_), or cycloheximide (CHX, 20 μg/mL). Glycolysis inhibition was tested via addition of 2-DG (10 or 50 mM). Glucose starvation was tested via treatment with glucose-containing (+ , 25 mM) or glucose-free (−, 0 mM) DMEM. **a** After four hours of treatment, cells were processed via luminescent cell viability assay to assess relative ATP content. Average luminescence was calculated from three technical replicates (or six replicate samples for the untreated, DMOG only, or hypoxia only treatments) and normalized to the untreated average values (set to 100%). Values are displayed as normalized average ± SD. *p < 0.01, **p < 0.001 (two-tailed unpaired t-test). After 4 (**b**) or 24 (**c**) hours of treatment, puromycin (10 μg/mL) was added to cells 10 min before collection. Whole cell lysates were processed via immunoblotting and blots were probed for the indicated antibodies. Blots are representative of three independent experiments

In RCC4 cells, ATP levels were minimally impacted by 4 h of treatment, with the exception of high-dose 2-DG (Fig. 3a). These effects were reflected in global translation rates (Fig. 3b). Similar to HEK293A cells, HIF1α expression was relatively unaffected by any treatment except for cycloheximide. By 24 h of treatment, the effects on global translation became more apparent, particularly with combination treatments (Fig. 3c). DMOG treatment alone appeared to suppress HIF1α levels by 24 h. The addition of 2-DG or glucose free-media did not further decrease HIF1α expression as in HEK293A cells. Hypoxia alone or in combination with glucose deprivation did not appear to substantially suppress HIF1α levels. These results suggest that HIF1α expression appears to be particularly sensitive to prolonged DMOG treatment rather than glucose deprivation in RCC4 cells.

### Glucose deprivation alone suppresses HIF1α synthesis rates by four hours

In general, the extent of HIF1α suppression mirrored the extent of ATP and global translation rate decline, indicating a potential translational effect on HIF1α. Although glucose deprivation alone did not substantially impact total HIF1α levels in *VHL-/-* cells, we sought to directly detect actively-translating HIF1α in response to glucose deprivation. This was done by isolating HIF1α via immunoprecipitation following 2-DG or glucose-free media treatment and puromycin addition. Immunoprecipitates were processed via immunoblot and probed for puromycin to detect puromycin-labelled (i.e., actively-translating) HIF1α across treatments.

**Fig. 4:**
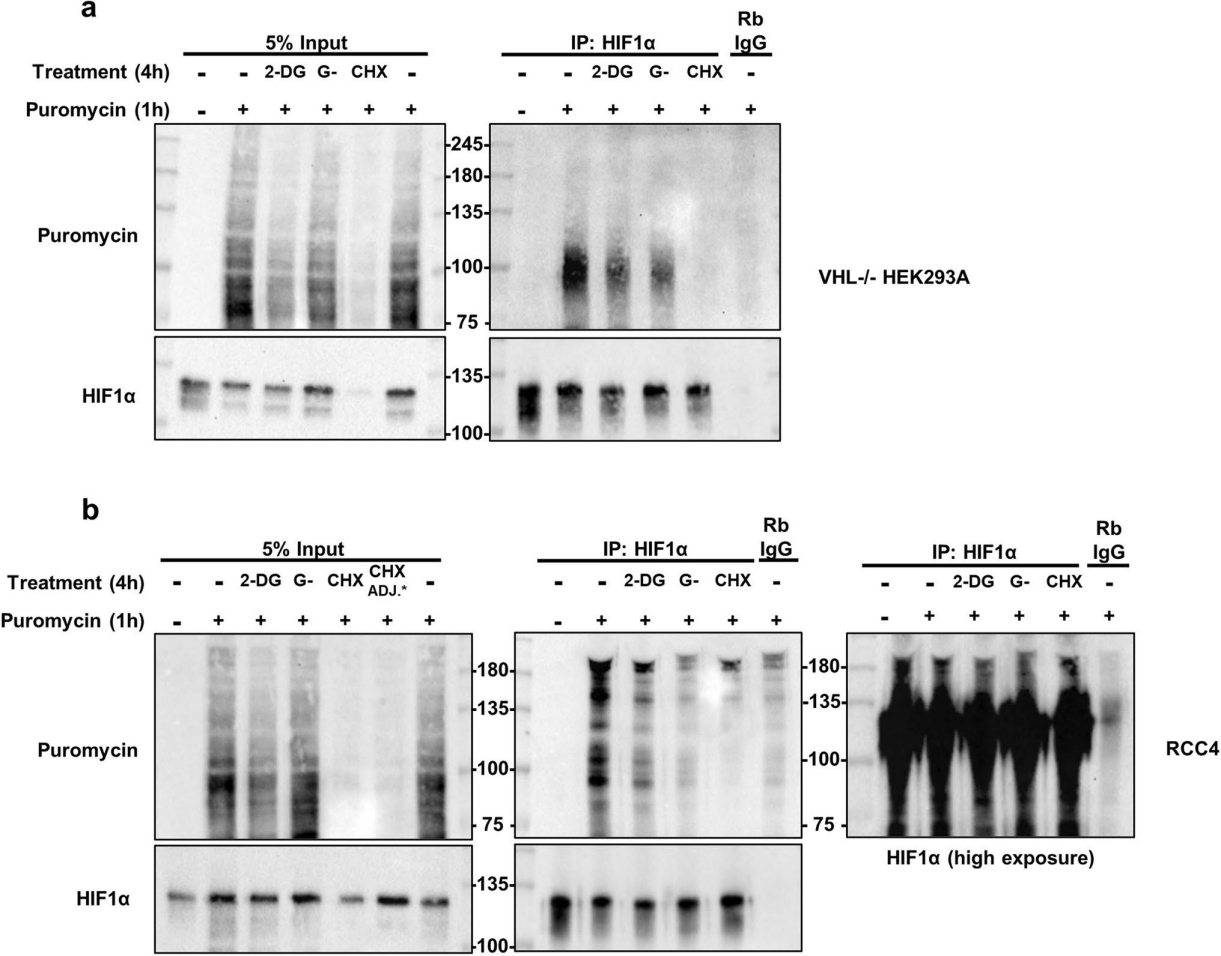
Glucose deprivation alone suppresses HIF1α synthesis rates in *VHL*-/- HEK293A cells and RCC4 cells. *VHL*-/- HEK293A cells (**a**) or RCC4 cells (**b**) were treated with 2-DG (10 mM), glucose-free DMEM (G-, 0 mM), or cycloheximide (CHX, 20 μg/mL) for four hours. Puromycin (10 μg/mL) was added to cells one hour before collection. Following HIF1α immunoprecipitation, immunoprecipitates (IP) and 5% lysate inputs were processed via immunoblotting and probed with the indicated antibodies. For RCC4 experiments in (**b**), extra whole-cell lysate was added in the cycloheximide-treated samples (CHX ADJ*) to ensure relatively equal pull-down of HIF1α. Blots are representative of three independent experiments

In *VHL-/-* HEK293A cells, 2-DG, glucose-free media, or cycloheximide treatment suppressed global translation rates to different extents by 4 h, as shown in the 5% inputs (Fig. 4a). Puromycin detection in HIF1α immunoprecipitates revealed a signal around 100 kDa in the puromycin-treated samples. Given that full-length HIF1α ranges from 100 to 120 kDa and that puromycin incorporation results in translation termination, this signal is consistent with what is expected for puro-HIF1α detection. The puro-HIF1α signal declined substantially with cycloheximide treatment, confirming that translation inhibition decreases puromycin-labelled (actively-translating) HIF1α levels. 2-DG (10 mM) or glucose-free media alone also appeared to decrease puro-HIF1α levels by 4 h. These results suggest that although total HIF1α levels appear to be relatively unaffected by glucose deprivation alone, HIF1α synthesis rates are suppressed by some extent with these treatments.

A similar experiment was performed in RCC4 cells. As expected, global translation rates declined slightly, or did not decline, with 2-DG or glucose-free media treatment, respectively (Fig. 4b). Puromycin detection in HIF1α immunoprecipitates also resulted in a signal in the puromycin-treated samples. This signal extended above the expected molecular weight of full-size HIF1α. However, the HIF1α signal in the immunoprecipitates created a near identical pattern at high molecular weights when overexposed, suggesting that this signal is likely puro-HIF1α. The puro-HIF1α signal decreased substantially with all treatments. As a result, HIF1α synthesis appears to also decline with glucose deprivation in RCC4 cells, despite little visible impact on global translation rates with glucose-free media treatment.

## Discussion

HIF transcription factors play a central role in the adaptive response to hypoxia [4]. However, dysregulation of HIFα can contribute to disease pathogenesis, including sporadic or inherited cancer syndromes [17]. Consequently, there has been much effort into understanding HIFα regulation. While the oxygen-dependent post-translational regulation of HIFα is well-characterized, there is evidence that HIFα expression responds to other cellular stimuli including reactive oxygen species, calcium signaling, oncogenic mutations, and nutrient availability [22, 33]. Rudimentary vascular systems within hypoxic tissues limit nutrient delivery, including glucose, to cells [30, 46]. This is further compounded by increased glucose consumption under hypoxia, leading to a risk of glucose deprivation [31, 46]. While the impacts of glucose deprivation on HIF1α expression have been explored previously, there are conflicting reports as to if this effect is at the translational or post-translational level.

Furthermore, the translational component of HIFα accumulation, particularly under cellular stress, is poorly understood. Hypoxia results in a biphasic downregulation of translation via the mTORC1 or PERK pathways [47]. Some mRNAs, including *HIF1A*, appear to be preferentially translated under hypoxia [48–50]. The exact mechanism behind the preferential translation of *HIF1A*, however, is still unclear [50]. It was argued that the *HIF1A* 5’ UTR possesses an internal ribosome entry site (IRES) that promotes cap-dependent translation under cellular stress [51]. The existence of a *HIF1A* IRES, however, has been met with controversy due to the unreliable nature of the bicistronic reporter genes assays used in these reports [52]. While it is evident that HIF1α accumulation is dependent on de novo protein synthesis, it is still unclear how HIF1α synthesis is maintained when global translation rates decrease [9, 49, 53]. Here, we show that limiting glucose availability via glycolysis inhibition or glucose starvation impairs HIF1α synthesis in HEK293A and RCC4 cells. In general, we found that decreased HIF1α synthesis coincided with a decline in global translation rates.

Our initial investigations in WT HEK293A cells found that glucose deprivation suppresses HIF1α accumulation by DMOG or hypoxia treatment, which is consistent with most previous reports [32–37]. The suppression of HIF1α appeared to be more evident with DMOG treatment, which may be due to the stronger overall stabilization of HIF1α by DMOG by 4 h compared to hypoxia. As expected, ATP levels and global translation rates declined modestly in response to glycolysis inhibition or glucose deprivation. DMOG or hypoxia co-treatment appeared to potentiate the effects of glucose deprivation on ATP levels and translation rates. This could be explained by the effects these treatments on cellular metabolism, which in turn impacts ATP generation and translation rates. Glucose deprivation, hypoxia, and DMOG have been shown to impair ATP production by decreasing glycolysis rates, oxidative phosphorylation, or inhibiting mitochondrial α-ketoglutarate-dependent hydroxylases, respectively [31, 54, 55]. When combined, the impacts of these treatments on ATP and translation rates are likely compounded. As a result, it was unclear if the effects on HIF1α levels were due to glucose deprivation alone, or combination with the treatments initially meant to stabilize HIF1α. For this reason, we used VHL-deficient cell lines to test the impact of glucose deprivation alone or in combination with DMOG or hypoxia on HIF1α expression.

Experimentation with *VHL-/-* HEK293A cells suggested that the combination of glucose deprivation with DMOG or hypoxia is required for HIF1α suppression. 2-DG or glucose starvation alone did not substantially decrease HIF1α levels, even by 24 h. Further, high-dose 2-DG did not suppress HIF1α levels to the same extent as combination treatments, despite decreasing ATP and translation rates to similar levels. However, puromycin-labelling experiments demonstrated that 2-DG or glucose starvation alone appears to decrease actively-translating HIF1α levels by 4 h. It is possible that little effect was seen on total HIF1α levels with glucose deprivation, even by 24 h, because of the large pool of stable HIF1α present in *VHL-/-* cells. Although we did not directly test combination treatments on HIF1α synthesis rates, the additional stress of DMOG or hypoxia may lead to a stronger translational suppression of HIF1α and a more rapid decline of total HIF1α levels.

In HEK293A cells, the decline in HIF1α synthesis rates coincided with decreased global translation rates, indicating this effect is not necessarily HIF1α-specific. In RCC4 cells, however, HIF1α synthesis rates declined substantially following glucose-free media treatment despite no apparent decline in global translation rates. These results suggest that at least in RCC4 cells, HIF1α synthesis may be more sensitive to glucose starvation than other actively-translating proteins. In this study, we found that HIF1α is not preferentially translated following global downregulation of protein synthesis via glucose deprivation. This differs from past reports of selective HIF1α synthesis under hypoxic translation downregulation [48–50]. Our results indicate that the preferential translation of HIF1α is likely specific to hypoxia and not other cellular stresses that downregulate global translation. Most research on HIFα synthesis focuses on the HIF1α paralog [50]. Comparatively little is known about HIF2α translation regulation. However, it has been shown that HIF2α synthesis is limited by iron availability via iron-responsive elements within *EPAS1* mRNA [56, 57]. As a result, HIF2α synthesis could possibly also respond to glucose availability in a similar manner as demonstrated in this study.

**Fig. 5:**
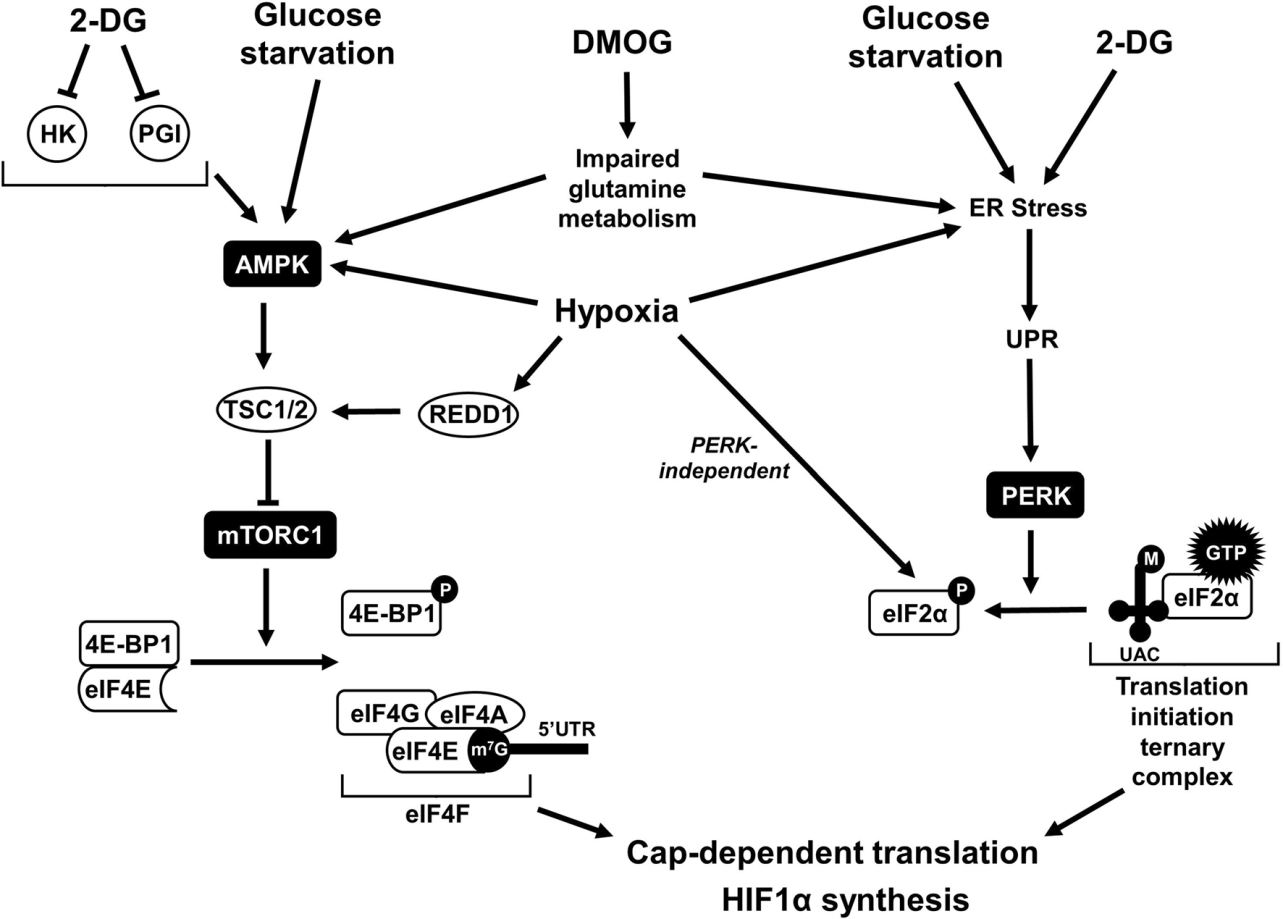
Schematic of how glycolysis inhibition, glucose starvation, hypoxia, and DMOG treatment may lead to global and HIF1α-specific translation downregulation. Eukaryotic translation initiation is mediated by two major regulatory complexes. Eukaryotic translation initiation factor (eIF) 4F consists of a cap-binding protein (eIF4E), helicase (eIF4A), and scaffolding protein (eIF4G) that associates with the 7-methylguanylate (m^7^G) cap on the 5’ untranslated region (UTR) of mature mRNA. The activity of eIF4F is regulated by mammalian target of rapamycin complex 1 (mTORC1), which promotes eIF4E-binding protein 1 (4E-BP1) phosphorylation when active. Increased AMP/ATP ratios lead to the activation of AMP-activated protein kinase (AMPK), inhibition of mTORC1, and hypophosphorylation of 4E-BP1. eIF4E is sequestered by hypophosphorylated 4E-BP1, resulting in decreased translation initiation. The translation initiation ternary complex consists of eIF2α, GTP, and initiator methionine tRNA. Endoplasmic reticulum (ER) stress leads to the activation of protein kinase R-like ER kinase (PERK) via the unfolded protein response (UPR), leading to eIF2α phosphorylation and deactivation. 2-deoxyglucose (2-DG) inhibits the glycolytic enzymes hexokinase (HK) and phosphoglucoisomerase (PGI), resulting in ATP decline and translational suppression. Similarly, glucose starvation impairs ATP generation. Both glycolysis inhibition and glucose starvation can promote protein misfolding and ER stress. Additionally, 2-DG has been shown to directly interfere with protein glycosylation, leading to ER stress and translational downregulation. Hypoxia leads to impaired ATP production and AMPK activation. Additionally, regulated in development DNA damage response 1 (REDD1) is transcriptionally upregulated by HIF1α, leading to mTORC1 inhibition by tuberous sclerosis complex 1 and 2 (TSC1/2) in prolonged hypoxia. Hypoxia also activates PERK via ER stress and may also promote PERK-independent eIF2α phosphorylation under prolonged hypoxia. Dimethyloxaloglycine (DMOG) is an α-ketoglutarate analog often used to promote HIF1α stabilization. There is evidence that DMOG interferes with glutamine metabolism, which could lead to eIF4F and eIF2α deactivation via the mTORC1 and PERK pathways, respectively. Figure created with Microsoft Powerpoint

HIF1α levels appeared to decline with prolonged DMOG treatment, particularly in RCC4 cells. Addition of 2-DG or glucose-free media did not further suppress HIF1α levels as in HEK293A cells. These results indicate that RCC4 cells may be particularly sensitive to DMOG treatment, potentially due to its metabolic effects. Renal cancer cells have been shown to depend on glutamine for survival [58]. As an α-ketoglutarate analog, DMOG may interfere with glutamine metabolism and promote ER stress [55, 59, 60], leading to the effects seen on global translation and HIF1α levels by 24 h (Fig. 5). Moreover, previous studies have shown that glutamine starvation impairs HIF1α accumulation similar to glucose starvation [33]. Future studies could clarify if the effects of DMOG are translational and due to interference with glutamine metabolism.

Studies have shown that 2-DG and glucose starvation can impair translation through two major pathways involving eukaryotic translation initiation factors (eIFs): the ATP-sensing mTORC1/eIF4E pathway, or the ER-mediated PERK/eIF2α pathway (Fig. 5) [31, 40–42]. It has been demonstrated that interference with both pathways can suppress HIF1α synthesis [28, 29, 61, 62]. The effects of 2-DG in this context, however, are likely not due to interruptions in N-glycosylation. We found that mannose addition (previously demonstrated to relieve ER stress [42]) did not rescue the suppression of translation and HIF1α levels by 2-DG (Supplementary Fig. 1).

Similarly, we attempted to clarify the role of ATP depletion in our results by providing a source of ATP downstream of 2-DG inhibition via pyruvate addition (Supplementary Fig. 2). As expected, exogenous pyruvate resulted in a near complete rescue in ATP to DMOG-only levels. Global translation rates increased modestly with pyruvate addition, but not to levels observed in DMOG-only treated cells. There were negligible changes in total HIF1α levels, suggesting that the slight increase in translation rates is insufficient to impact HIF1α levels. These observations suggest that global translation rates may lag behind the increase in ATP levels under these experimental conditions.

It is unclear why translation rates are marginally impacted despite recovery of ATP levels back to DMOG-only levels. One possibility is that pyruvate, as well as other glycolytic intermediates, have been shown to induce ER stress [63], which could lead to translation suppression. Thus, if HIF1α is sensitive to pyruvate-induced ER stress, this would mask the increase in global translation and HIF1α levels expected from the ATP recovery by exogenous pyruvate.

## Concluding remarks

The present study demonstrates that maximal HIF1α accumulation is dependent on glucose availability, adding to a growing field of non-canonical HIFα regulatory mechanisms. By directly measuring HIF1α synthesis in VHL-deficient cell lines, our results exclude the possibility of pVHL-dependent degradation of HIF1α by glucose deprivation, as previously suggested [35, 39], and support the notion that the consequential ATP depletion contributes, at least in part, to reduced global and HIF1α-specific translation. The sensitivity of HIF1α to glucose could be a useful target for metabolic therapy, particularly in cancer treatment [31, 64]. Future explorations could focus on the role of other essential nutrients on HIF1α regulation and understanding the relationship between HIF1α synthesis and global translation under metabolic stress.

## Supplementary Information

Supplementary material 1

Supplementary material 2

Supplementary material 3

## Data Availability

All data supporting the findings of this study are available within the paper and its Supplementary Information.
