# Supplementary material for: Glucose deprivation impairs hypoxia-inducible factor-1α synthesis
Source: Discov Oncol. 2024 Oct 28;15:595. doi: 10.1007/s12672-024-01484-1 (PMC11519269; doi:10.1007/s12672-024-01484-1)
Supplement: Supplementary file 1 — Supplementary material 1 [file 12672_2024_1484_MOESM1_ESM.docx]

**Supplementary Material**

Journal: Discover Oncology

**Hypoxia-inducible factor-1α synthesis rates are impaired under glucose deprivation**

Mia Hubert^1^, Sarah Stuart^1,2^, Michael Ohh^1,2^

1 Department of Laboratory Medicine & Pathobiology, Faculty of Medicine, University of Toronto, 1 King’s College Circle, Toronto, Ontario, Canada, M5S 1A8

2 Department of Biochemistry, Faculty of Medicine, University of Toronto, 1 King’s College Circle, Toronto, Ontario, Canada, M5S 1A8

Correspondence to: Prof. Michael Ohh; Email: [michael.ohh@utoronto.ca](mailto:michael.ohh@utoronto.ca); Tel: +1 (416) 946-7922

**
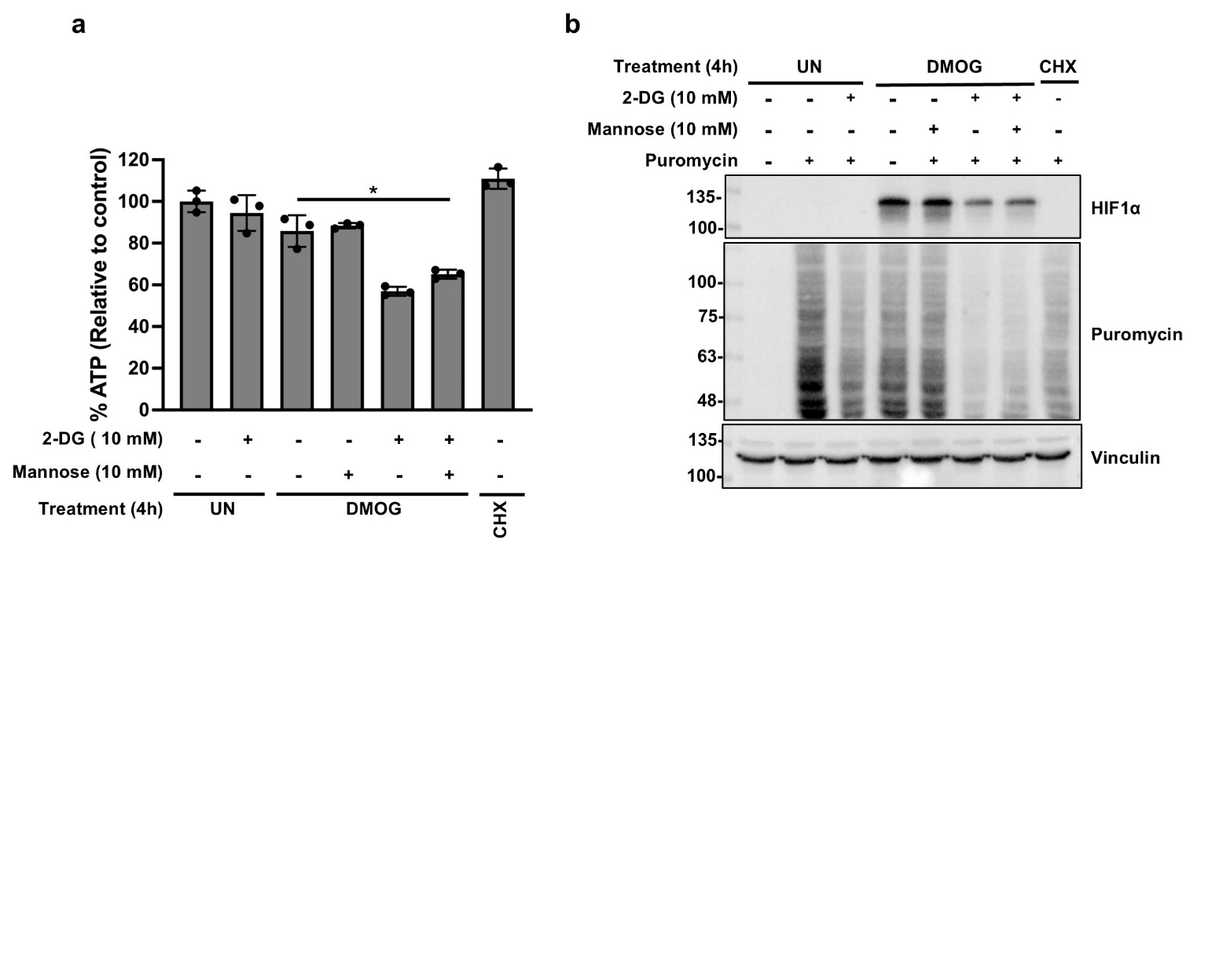
**

**Supplementary Fig. 1** Mannose addition does not rescue the 2-DG-mediated suppression of global translation rates or HIF1α levels. In all experiments, WT HEK293A cells were treated with DMOG (1 mM) to stabilize HIF1α, 2-DG (10 mM) ± mannose (10 mM), or cycloheximide (CHX, 20 μg/mL). (a) After four hours of treatment, cells were processed via luminescent cell viability assay to assess relative ATP content. Average luminescence was calculated from three technical replicates and normalized to the untreated average values (set to 100%). Values are displayed as normalized average ±SD. Mannose addition did not rescue ATP back to DMOG-only levels (* indicates p < 0.01, two-tailed unpaired t-test). (b) After 4 hours puromycin (10 μg/mL) was added to cells 10 minutes before collection. Whole cell lysates were processed via immunoblotting and blots were probed for the indicated antibodies. Blots are representative of three independent experiments


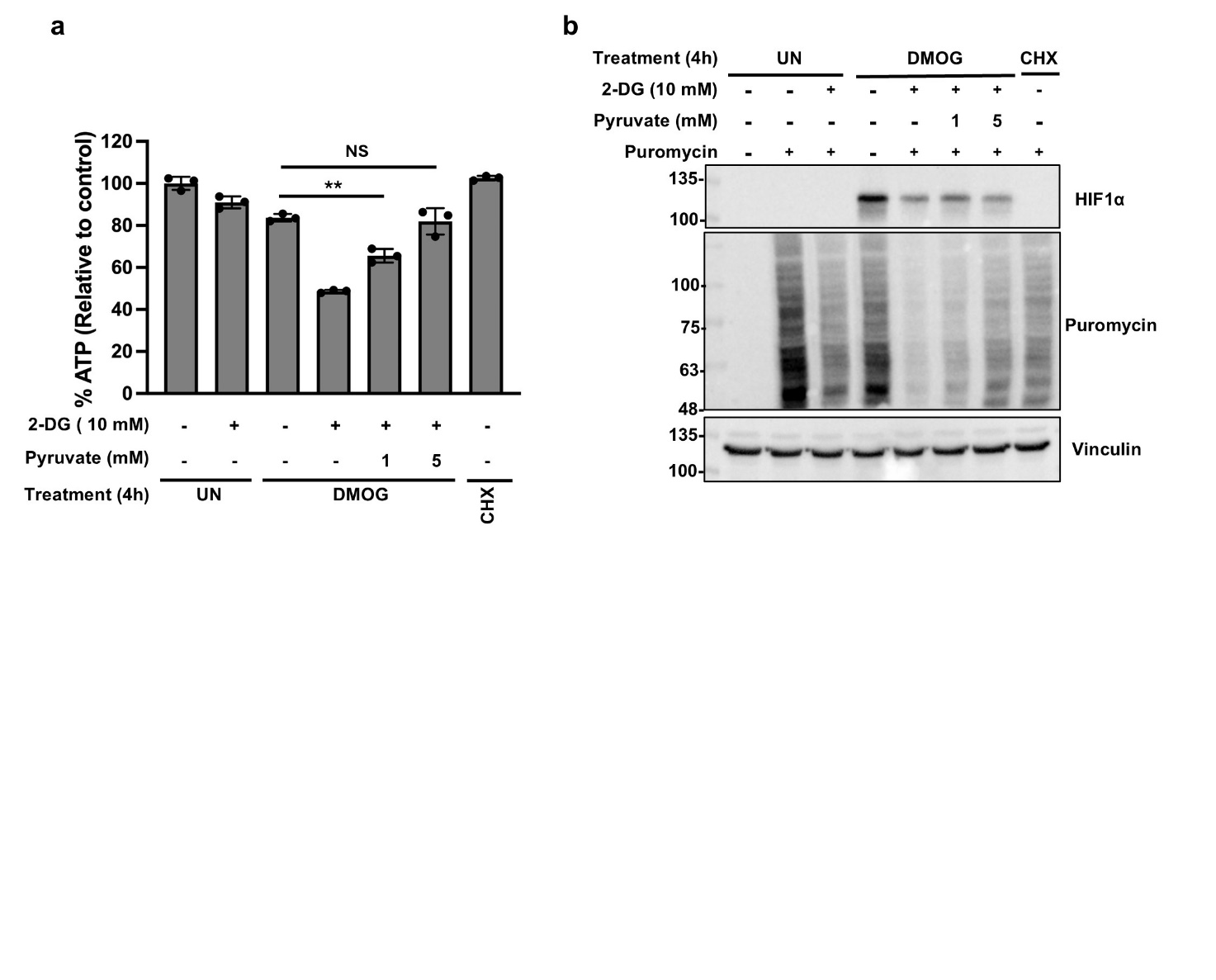


**Supplementary Fig. 2** Pyruvate addition rescues ATP levels but not global translation rates or HIF1α levels. WT HEK293A cells were treated with DMOG (1mM) to stabilize HIF1α in addition to 2-DG (10 mM) +/- Na-pyruvate (1 or 5 mM) for 4 hours. Cycloheximide (20 μg/mL) treatment was included as a positive control for translation downregulation. (a) Cells were processed via luminescence-based viability assays to monitor ATP levels. Average luminescence was calculated from three technical replicates and normalized to the untreated average values (set to 100%). Values are displayed as normalized average ±SD. Pyruvate-treated samples were statistically compared to DMOG-only levels (** indicates p < 0.001, NS = non significance, two-tailed unpaired t-test). (b) Cells were processed via Western blot to monitor translation and HIF1α levels. Puromycin (10 μg/mL) was added to Western blot samples 10 minutes before collection. Blots are representative of three independent experiments
