# Supplementary material for: Glucose deprivation impairs hypoxia-inducible factor-1α synthesis
Source: Discov Oncol. 2024 Oct 28;15:595. doi: 10.1007/s12672-024-01484-1 (PMC11519269; doi:10.1007/s12672-024-01484-1)
Supplement: Supplementary file 3 — Supplementary material 3 [file 12672_2024_1484_MOESM3_ESM.docx]

**Supplementary Material**

Journal: Discover Oncology

**Hypoxia-inducible factor-1α synthesis rates are impaired under glucose deprivation**

Mia Hubert^1^, Sarah Stuart^1,2^, Michael Ohh^1,2^

1 Department of Laboratory Medicine & Pathobiology, Faculty of Medicine, University of Toronto, 1 King’s College Circle, Toronto, Ontario, Canada, M5S 1A8

2 Department of Biochemistry, Faculty of Medicine, University of Toronto, 1 King’s College Circle, Toronto, Ontario, Canada, M5S 1A8

Correspondence to: Prof. Michael Ohh; Email: [michael.ohh@utoronto.ca](mailto:michael.ohh@utoronto.ca); Tel: +1 (416) 946-7922

**Unprocessed Blots from Figures 1-4 and Supplementary Figures 1-2**

Blots were labelled and annotated with Microsoft PowerPoint to create final figures. Red boxes indicate where images were cropped to create final figures. Detected proteins are indicated in text beside the blots. Blots are in order from top to bottom, left to right.

**Figure 1, panel a**


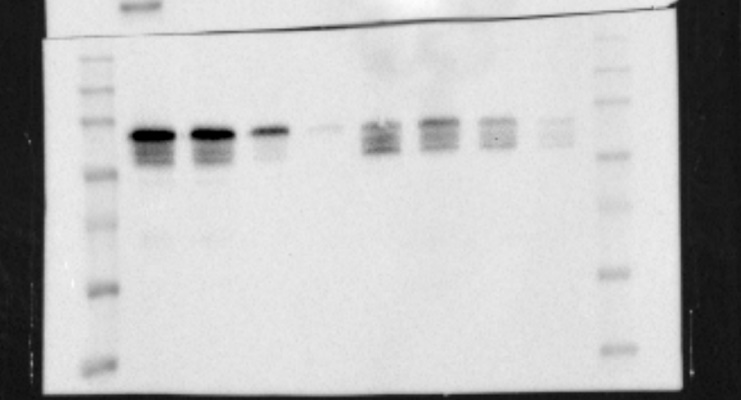
 HIF1α


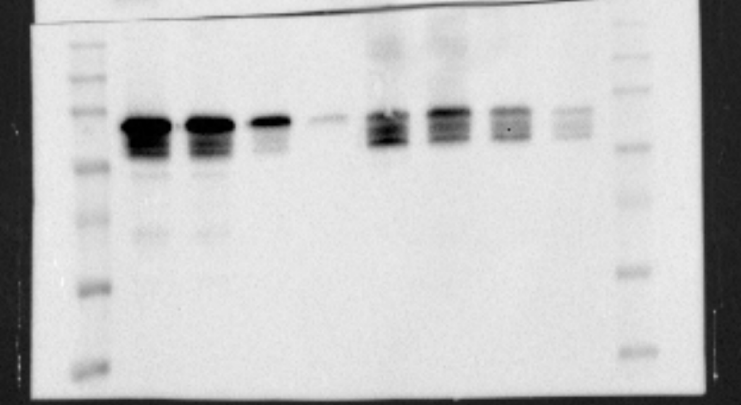
 HIF1α (high exposure)


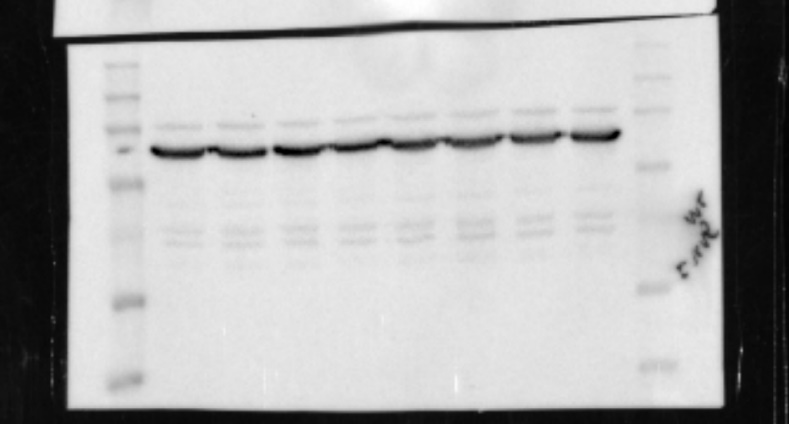
 Vinculin

**Figure 1, panel b** (rotated images slightly due to slanted blot)


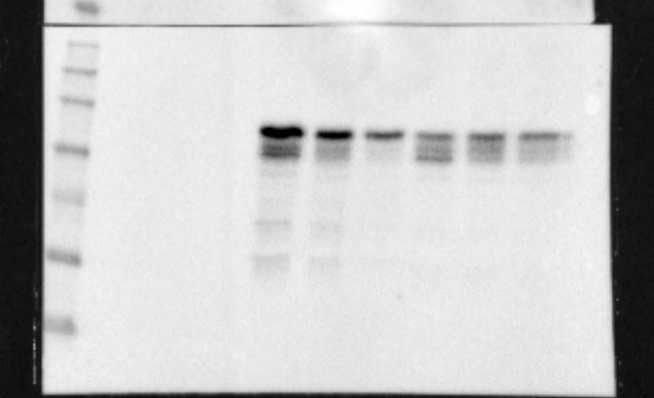
 HIF1α


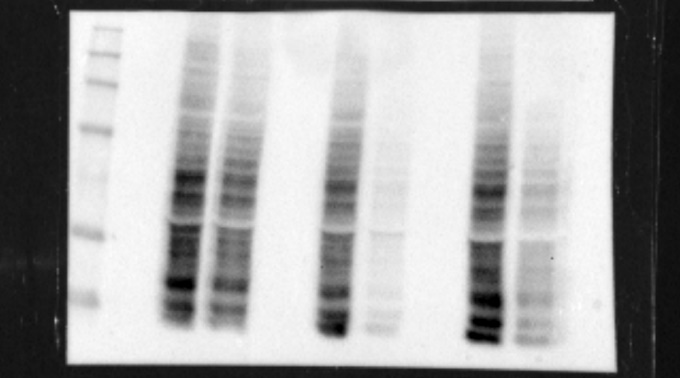
 Puromycin


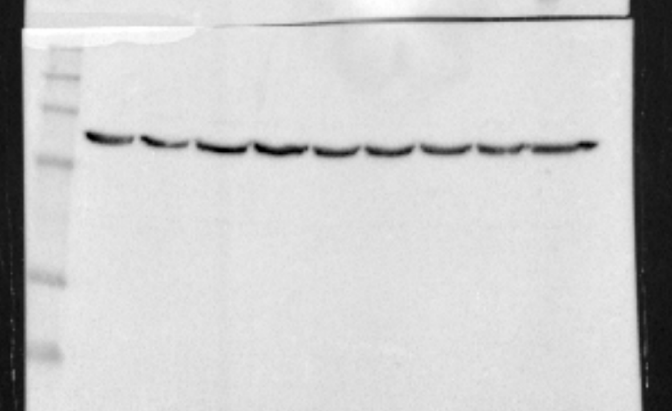
 Vinculin

**Figure 1, panel c**


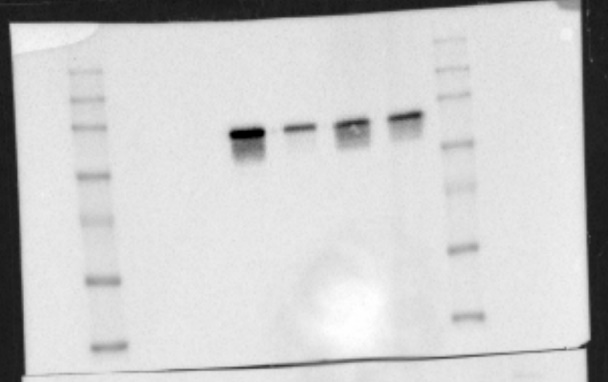
 HIF1α


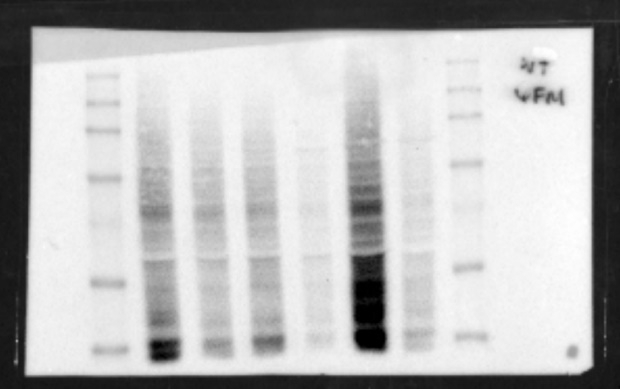
 Puromycin


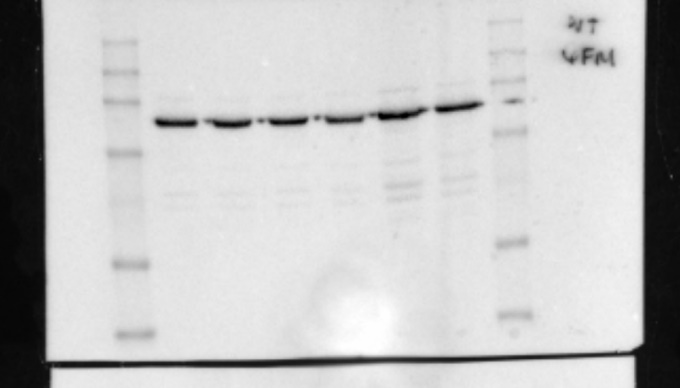
 Vinculin

**Figure 2, panel b, blot 1** (2-DG treatment, 4h)


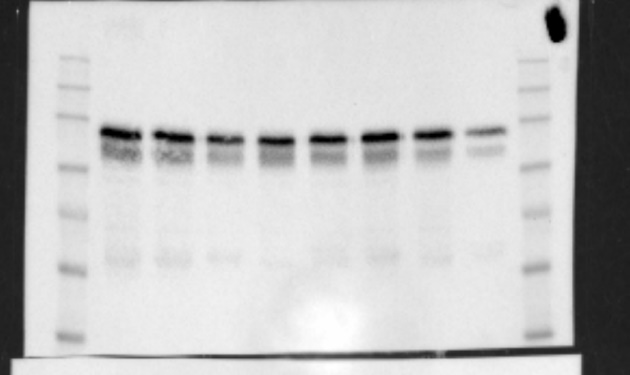
 HIF1α


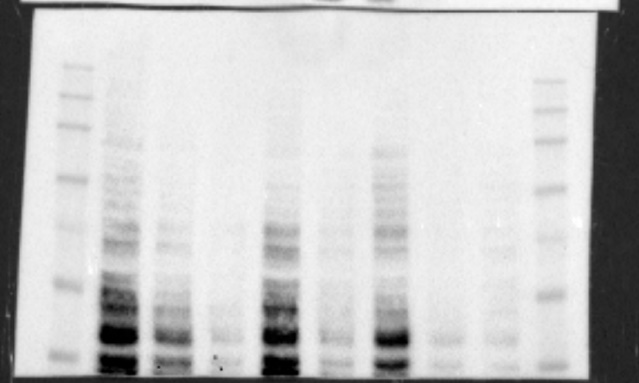
 Puromycin


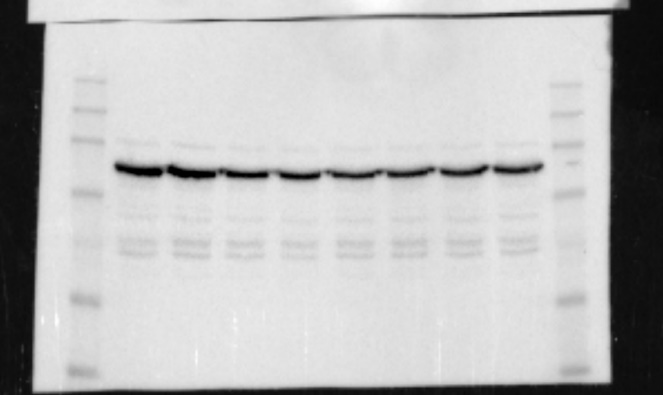
 Vinculin

**Figure 2, panel b, blot 2** (glucose-free media treatment, 4h)


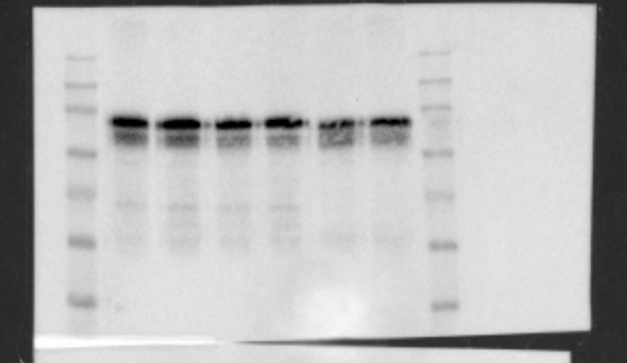
 HIF1α


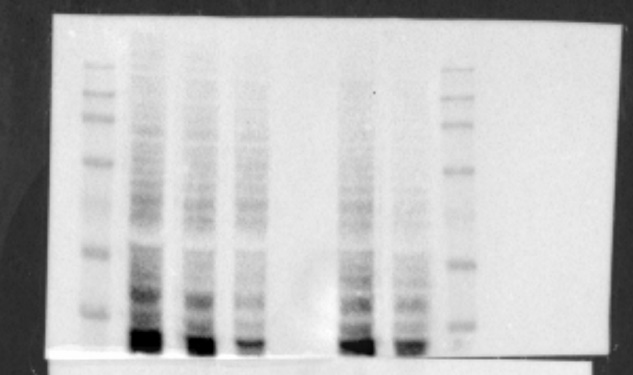
 Puromycin


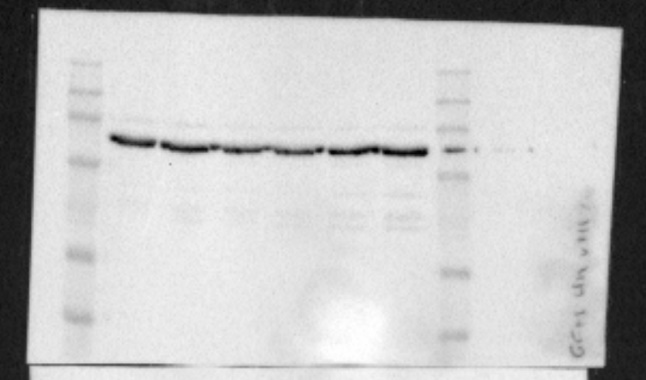


**Figure 2, panel c, blot 1** (2-DG treatment, 24h)


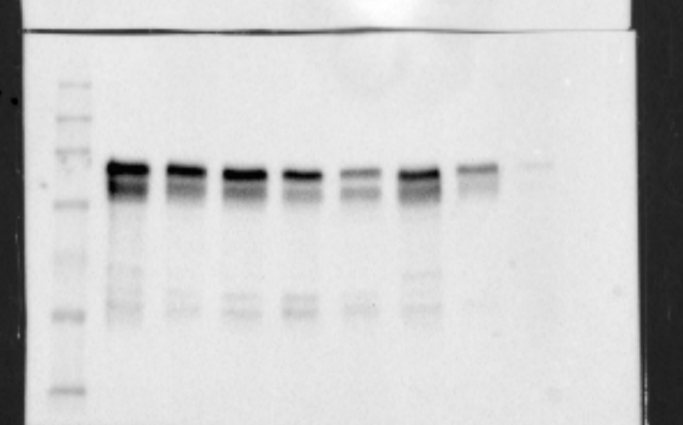
 HIF1α


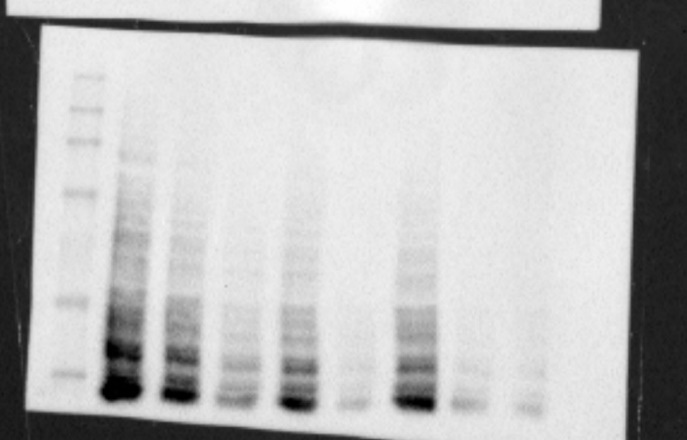
 Puromycin


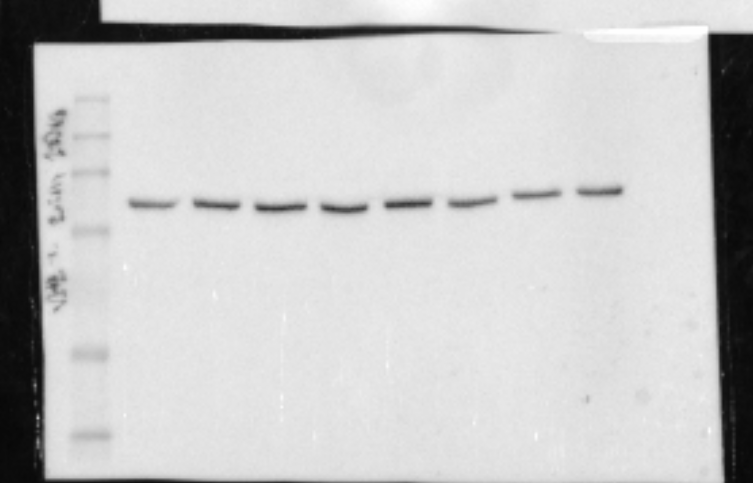
 Vinculin

**Figure 2, panel c, blot 2** (glucose-free media treatment, 24h)


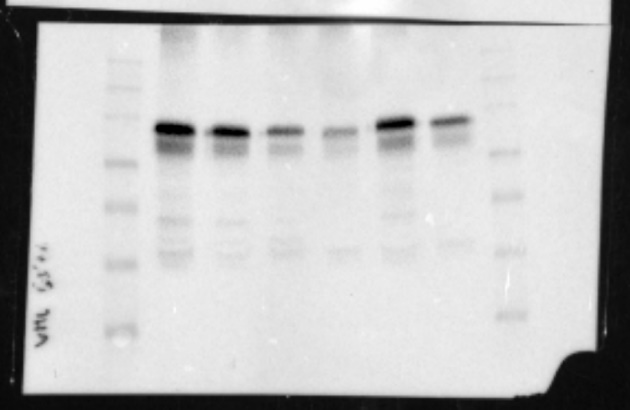
 HIF1α


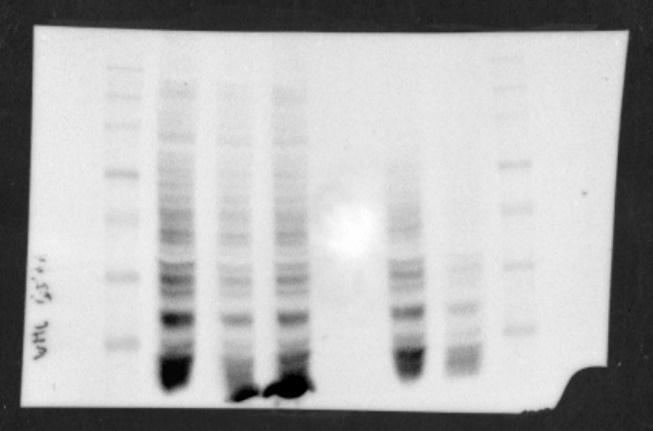
 Puromycin


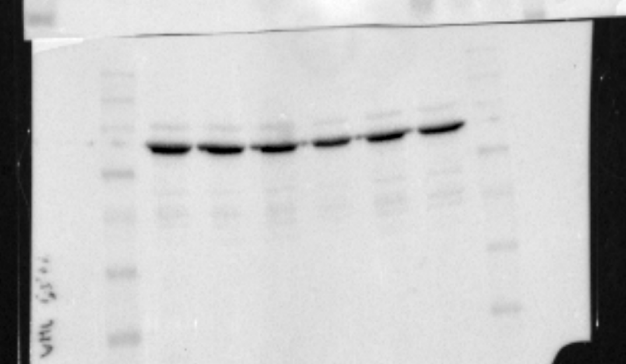
 Vinculin

**Figure 3, panel b, blot 1** (2-DG treatment, 4h)


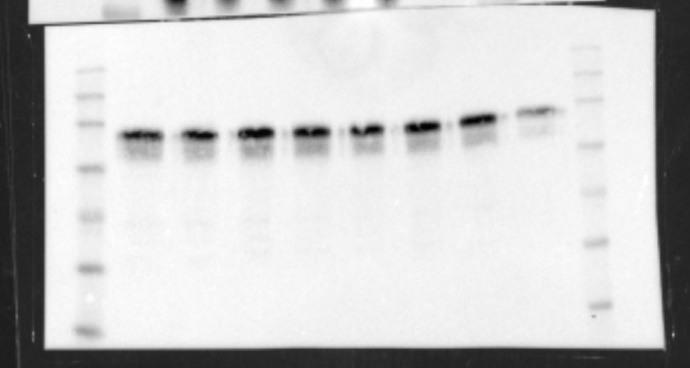
 HIF1α


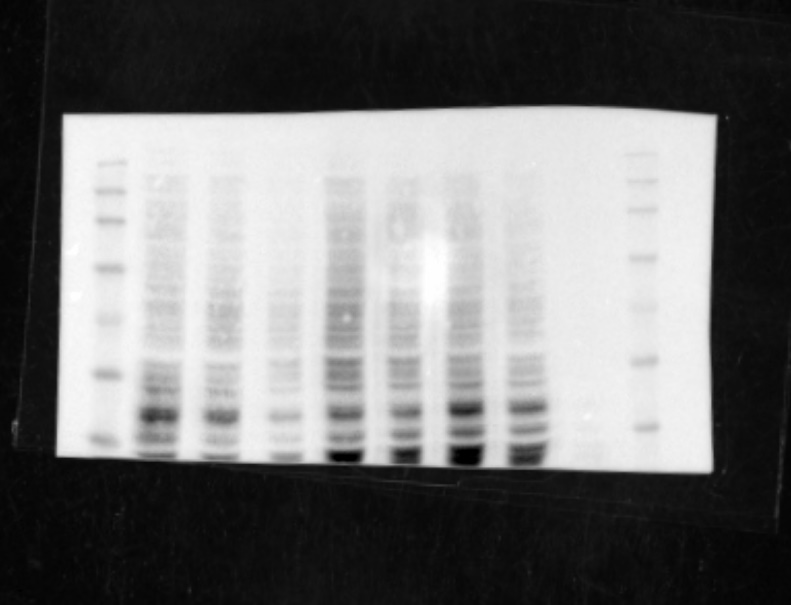
 Puromycin


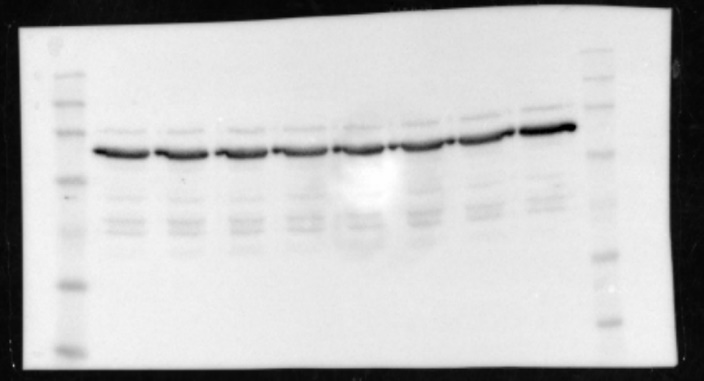
 Vinculin

**Figure 3, panel b, blot 2** (glucose-free media treatment, 4h)


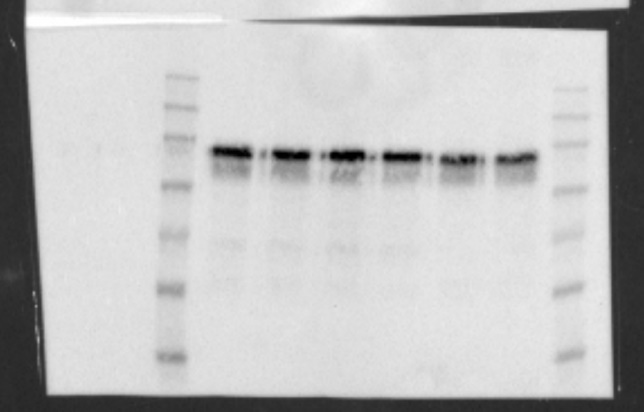
 HIF1α


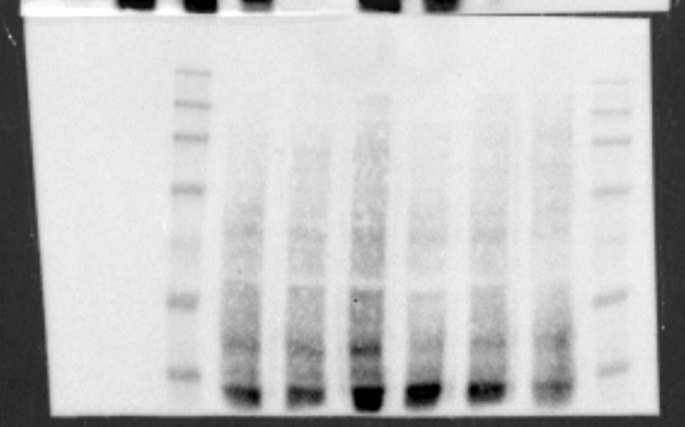
 Puromycin


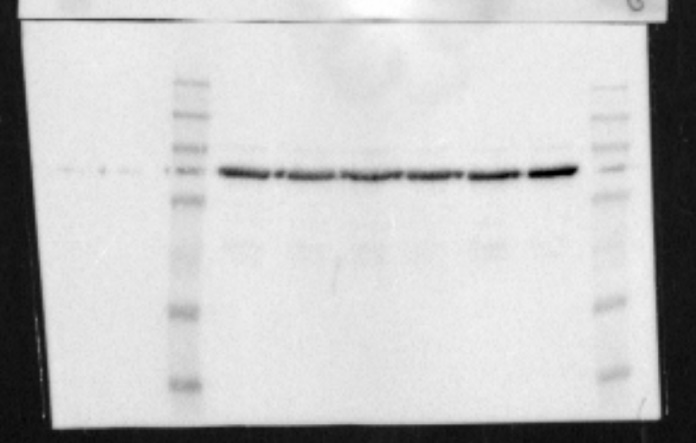
 Vinculin

**Figure 3, panel c, blot 1** (2-DG treatment, 24h)


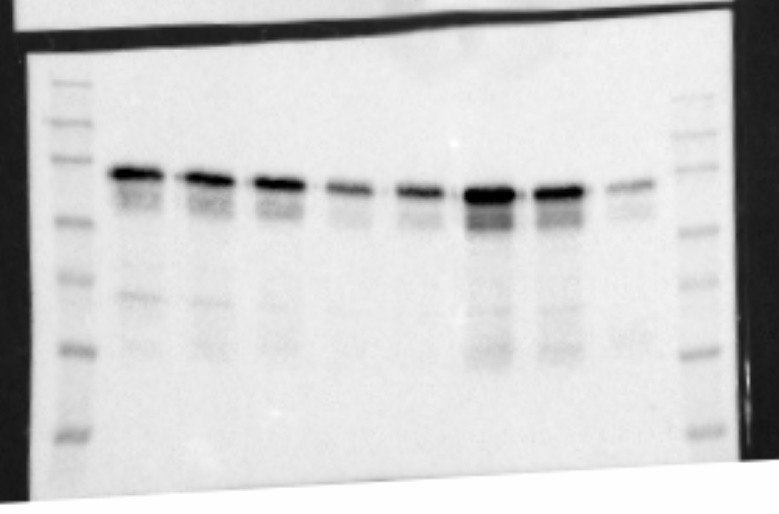
 HIF1α


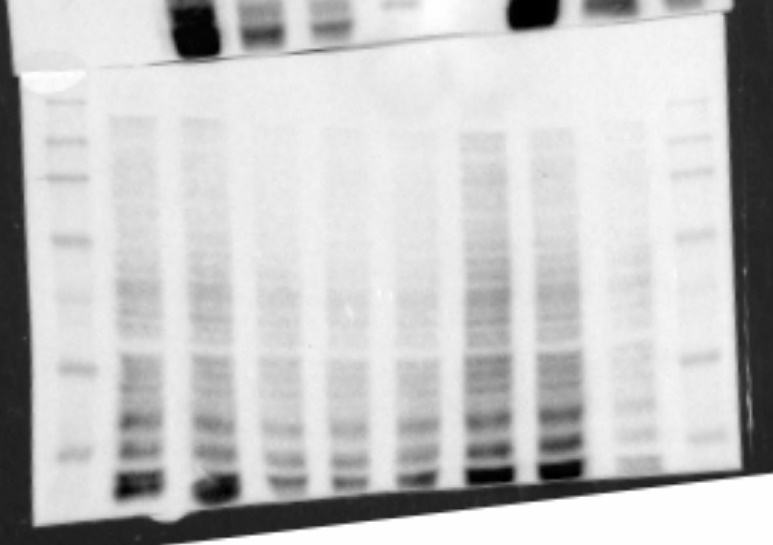
 Puromycin
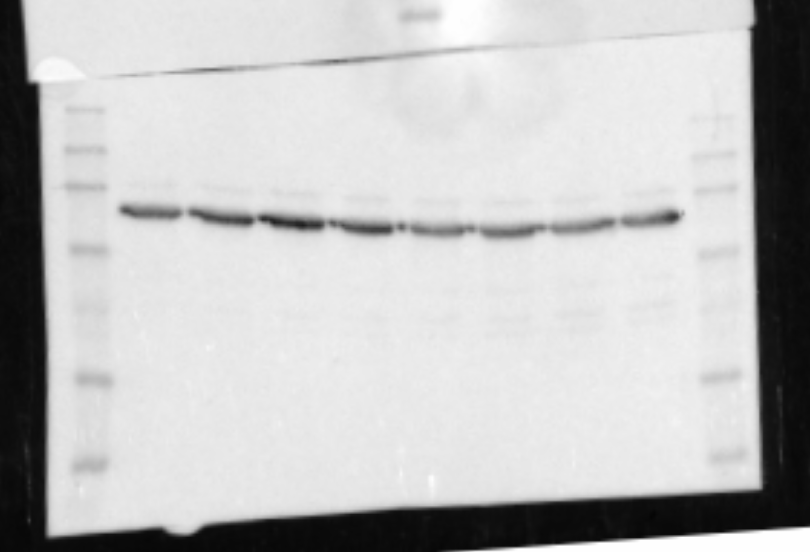
 Vinculin

**Figure 3, panel c, blot 2** (glucose-free media treatment, 24h)


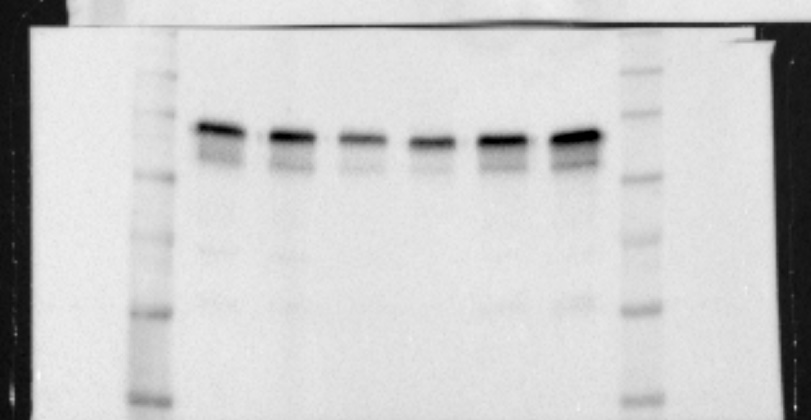
 HIF1α
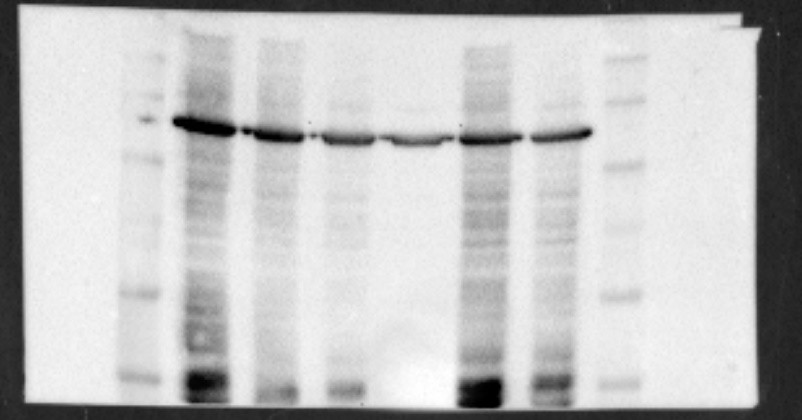
 Puromycin (upper bands = residual vinc signal)
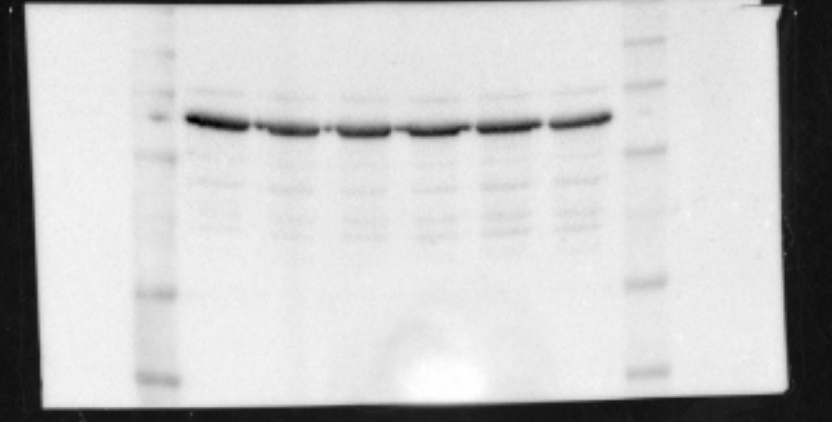
 Vinculin

**Figure 4, panel a**


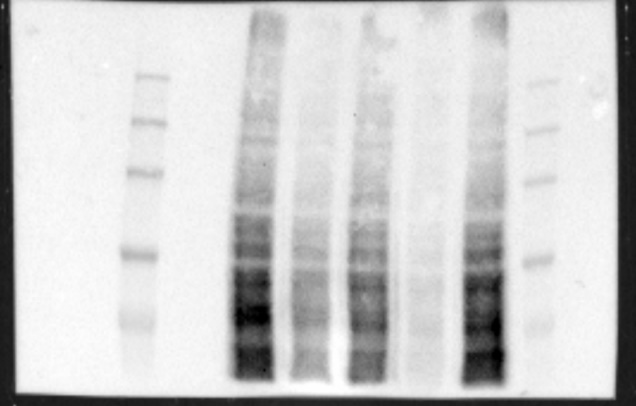
 Puromycin (5% input blot)


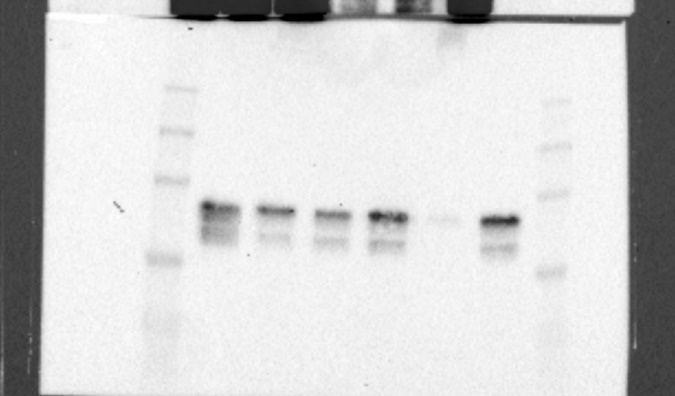
 HIF1α (5% input blot)


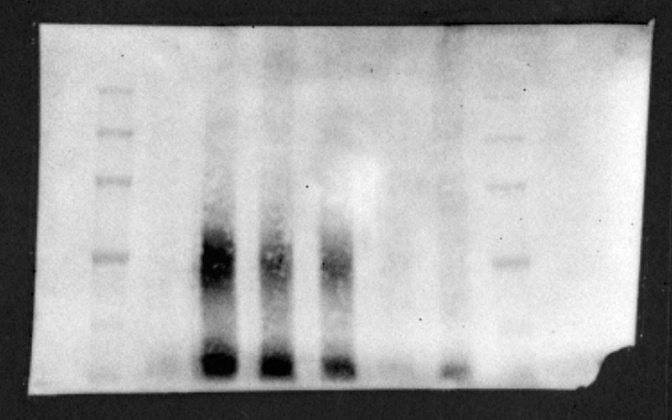
 Puromycin (IP blot)


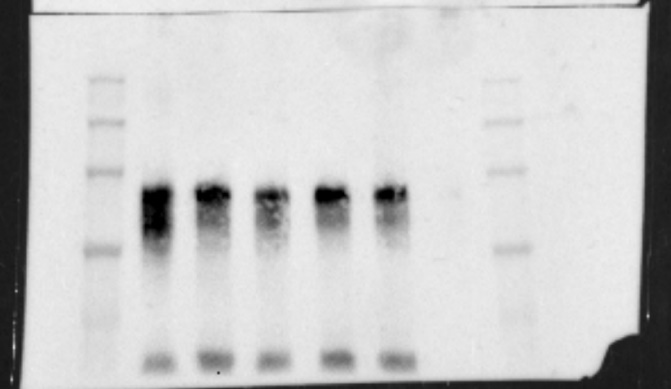
 HIF1α (IP blot)

**Figure 4, panel b**


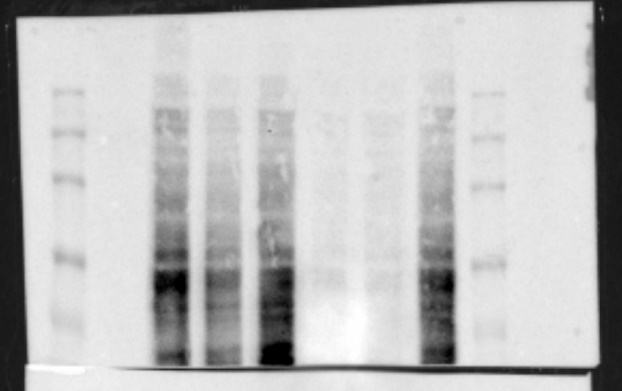
 Puromycin (5% input blot)


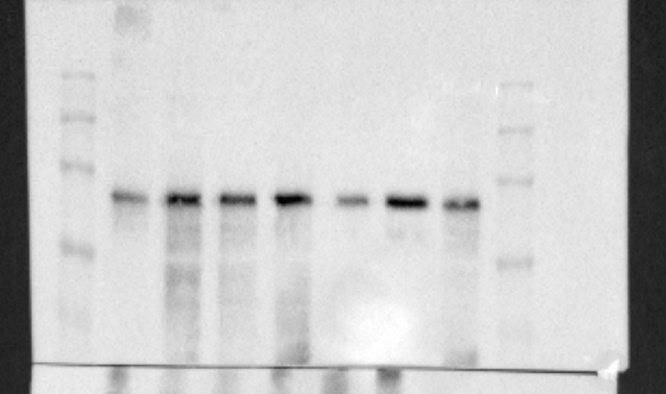
 HIF1α (5% input blot)


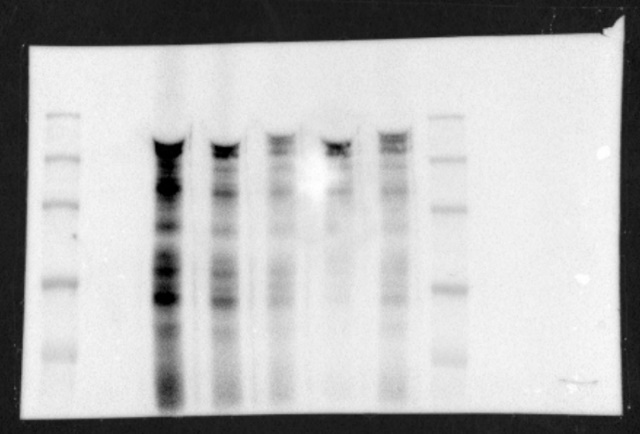
 Puromycin (IP blot)


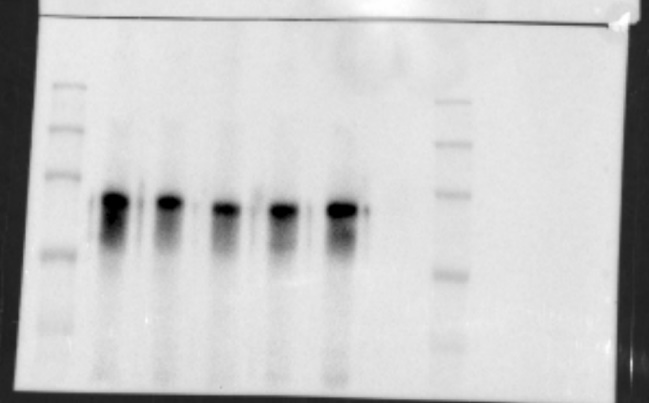
 HIF1α (IP blot)


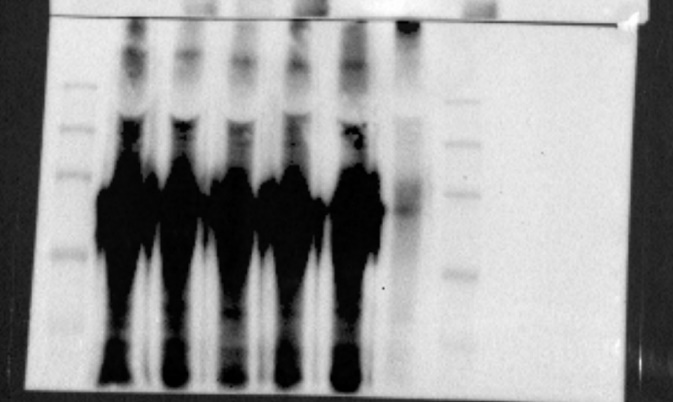
 HIF1α, high exposure (IP blot)

**Supplementary Figure 1**

**
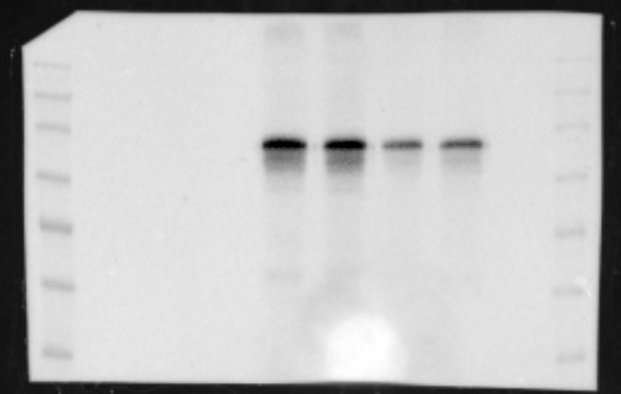
** HIF1α

**
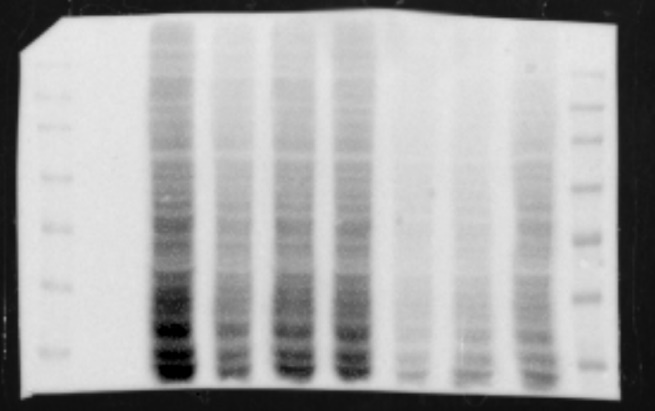
**Puromycin

**
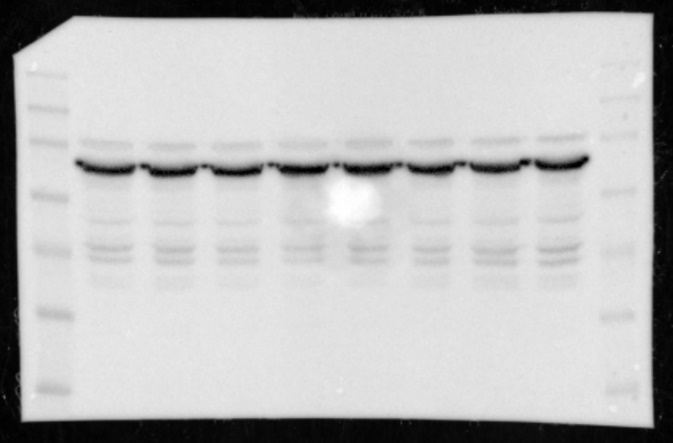
** Vinculin

**Supplementary Figure 2**

**
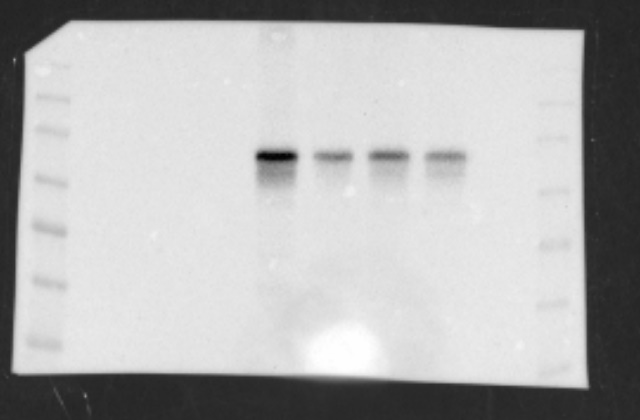
** HIF1α

**
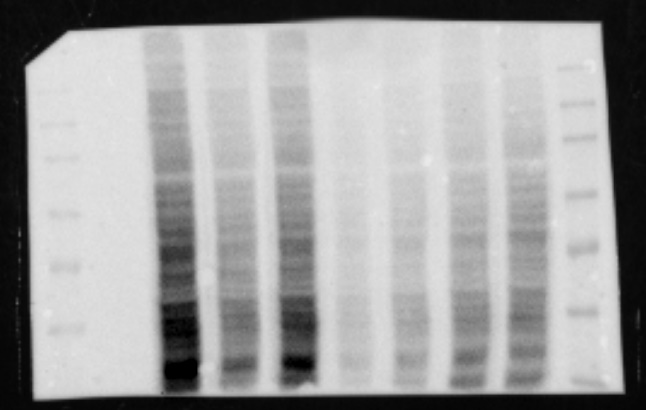
** Puromycin

**
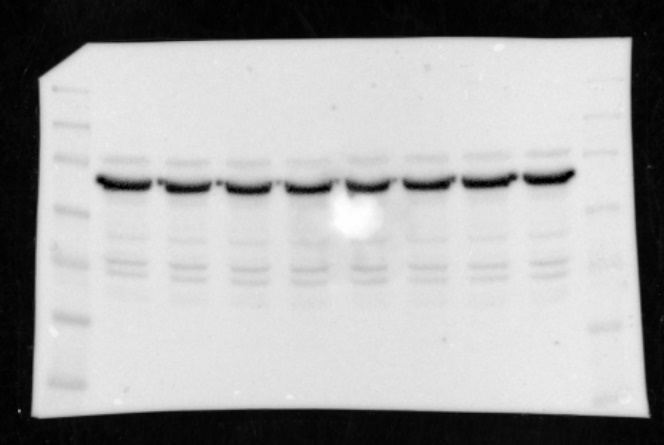
** Vinculin
